# A xenotransplantation mouse model to study physiology of the mammary gland from large mammals

**DOI:** 10.1371/journal.pone.0298390

**Published:** 2024-02-28

**Authors:** James L. Miller, Alexandra Reddy, Rebecca M. Harman, Gerlinde R. Van de Walle

**Affiliations:** Baker Institute for Animal Health, College of Veterinary Medicine, Cornell University, Ithaca, New York, United States of America; The University of Queensland Faculty of Medicine, AUSTRALIA

## Abstract

Although highly conserved in structure and function, many (patho)physiological processes of the mammary gland vary drastically between mammals, with mechanisms regulating these differences not well understood. Large mammals display variable lactation strategies and mammary cancer incidence, however, research into these variations is often limited to *in vitro* analysis due to logistical limitations. Validating a model with functional mammary xenografts from cryopreserved tissue fragments would allow for *in vivo* comparative analysis of mammary glands from large and/or rare mammals and would improve our understanding of postnatal development, lactation, and premalignancy across mammals. To this end, we generated functional mammary xenografts using mammary tissue fragments containing mammary stroma and parenchyma isolated via an antibody-independent approach from healthy, nulliparous equine and canine donor tissues to study these species *in vivo*. Cryopreserved mammary tissue fragments were xenotransplanted into de-epithelialized fat pads of immunodeficient mice and resulting xenografts were structurally and functionally assessed. Preimplantation of mammary stromal fibroblasts was performed to promote ductal morphogenesis. Xenografts recapitulated mammary lobule architecture and contained donor-derived stromal components. Mammatropic hormone stimulation resulted in (i) upregulation of lactation-associated genes, (ii) altered proliferation index, and (iii) morphological changes, indicating functionality. Preimplantation of mammary stromal fibroblasts did not promote ductal morphogenesis. This model presents the opportunity to study novel mechanisms regulating unique lactation strategies and mammary cancer induction *in vivo*. Due to the universal applicability of this approach, this model serves as proof-of-concept for developing mammary xenografts for *in vivo* analysis of virtually any mammals, including large and rare mammals.

## Introduction

The mammary gland is a defining feature of all mammals and is conserved in function and structure. It is a specialized, hormone-responsive organ that develops postnatally and undergoes cycles of growth, differentiation, lactation, and involution, and is comprised of a branching ductal network that terminates in functional secretory alveolar structures when fully developed [1–3]. Despite its conserved nature, the mammary gland is highly variable across mammals regarding lactation strategy, disease incidence, and gross morphology, with glands from larger mammals displaying greater architectural complexity relative to more rudimentary glands in mice [4]. These variations allow for comparative species approaches to identify novel regulatory factors and underlying molecular mechanisms of mammary gland (patho)physiology that are relevant to both human and veterinary mammary health [4–7].

While *in vitro* models, such as mammary organoids, mimic 3D mammary gland architecture [8, 9], *in vivo* studies allow for studying mammary tissues within a vascularized supportive stromal environment as well as exposure to the complex hormonal milieu required for both normal and malignant mammary cell function [10, 11]. *In vivo* mammary studies, however, are difficult when assessing large and/or undomesticated mammals due to logistical challenges such as cost and/or limited availability. Thus, a manageable *in vivo* model is warranted. To this end, xenotransplantation models, i.e., immunodeficient laboratory mice containing functional tissues derived from other species, have notable value for *in vivo* tissue analysis, particularly since xenotransplanted tissues maintain a 3D architecture and are supported by host environmental factors. Historically, xenotransplantation models have served as valuable tools to understand cancer biology [12–14], assess stem/progenitor cell repopulation potential [15, 16], and resolve critical developmental mechanisms [17]. Furthermore, drug responses in xenotransplanted tissues, both healthy and malignant, often align with clinical responses [14, 18–20], indicating their value in modeling drug pharmacokinetics. Regarding the generation of mammary xenografts, one approach relies on the murine mammary fat pad de-epithelializing (clearing) method, in which the postnatally-developing endogenous mammary epithelium is excised during puberty, at about 3–4 weeks of age, followed by xenotransplantation of typically freshly obtained mammary tissues or sorted stem/progenitor cells from other mammals into the residual stroma [21], as has been described for rat [22], cow [15, 23–27], and human [28–30].

Here, we report on a reproducible and functional xenotransplantation model that uses cryopreserved mammary tissue fragments from equine or canine donors. These mammals were selected based on (i) availability of tissues from research animals, (ii) their status as sentinel species, i.e., large mammals that share human environments [7], (iii) similarities to humans regarding mammary morphology [31, 32], and (iv) variation in their natural mammary cancer incidence, with horses and dogs representing mammals with a low and high mammary cancer incidence, respectively [7, 33]. Importantly, the mammary tissue fragments used for xenotransplantation were isolated via an antibody-free mechanical and enzymatic digestion method, making this approach universally applicable to virtually any mammal. Moreover, the use of cryopreserved mammary tissue fragments for xenotransplantation allows for the processing and generation of large quantities of mammary tissue fragments for long term storage, which is critical for studies of rare and scarcely accessible wild mammals.

Our salient findings were that both equine and canine xenografts consisted of lobule-like mammary outgrowths, containing multiple acini, and recapitulated the cellular architecture of the donor mammary gland. Importantly, the xenografts responded to circulating murine mammotropic hormones, confirming tissue functionality. We propose this model using cryopreserved mammary gland tissue fragments to be appropriate for establishing mammary xenografts of virtually any mammal, especially those that are large and/or rare, and to represent the unique opportunity for physiology studies of the mammary gland of non-traditional, large mammals *in vivo* within the context of manageable laboratory mice.

## Materials and methods

### Ethics statement

All experimental procedures were performed in accordance with relevant guidelines and regulations and were approved by the Institutional Animal Care and Use Committee (IACUC) at Cornell University (#2013–0022). Tissues obtained from equine (*Equus caballus*) and canine (beagles, *Canis lupus familiaris*) donors were recovered after euthanasia. All donors were euthanized for reasons unrelated to this study and all tissues were extracted post-mortem, therefore, IACUC approval was not required for the mammary gland tissue collections.

### Generation and cryopreservation of mammary tissue fragments for xenotransplantation

Mammary gland tissues were recovered from clinically healthy, intact, virgin animals, that were euthanized for reasons unrelated to this study. Tissues were obtained from equine (*Equus caballus*) donors (various breeds) aged between 2 to 16 years, consisting of 5 cm^2^ equine mammary gland tissue pieces bordering the mammary gland compartments, and canine (*Canis lupus familiaris*) donors (beagles) aged ~ 15 months, consisting of 2 cm^2^ mammary gland tissue pieces from the mammary duct near the nipple. The large age range in equine tissue donors was due to the need to opportunistically obtain scare equine tissues for xenotransplantations, thus, tissues were extracted from animals of variable age. Notably, studies assessing multiple xenotransplanted tissue types have shown that donor age may impact xenograft function and behavior [34], however, it is well-acknowledged that mammary gland tissues from aged donors can still regenerate fully functional mammary glands [35]. All tissues were extracted at Cornell College of Veterinary Medicine (CVM) or at the Cornell Animal Health Diagnostic Center (AHDC). Reproductive history was validated by caretaker/veterinarian documentation records.

Mammary tissue fragments were generated as previously described [4, 36, 37]. Briefly, tissues were mechanically digested using surgical scissors and then enzymatically digested for 3 h at 37°C in a solution of Dulbecco’s modified Eagle’s medium (DMEM) (cat #: 10-014-CV) /Ham’s F12 (cat #: 10-080-CV) (Corning Inc., Sommerville, MA) (DMEM/F12) (50/50), containing collagenase (300 U/ml) (cat #: LS004176) (Worthington, Lakewood, NY), Hyaluronidase (100 U/ml) (cat #: H3506) (Sigma Aldrich, St. Louis, MO), 5% fetal bovine serum (FBS) (cat #: S11550) (R&D Systems, Minneapolis, MN), insulin (5 μg/ml) (cat #: 12585–014) (Sigma Aldrich), and hydrocortisone (1 μg/ml) (cat #: H2270) (Sigma Aldrich). Digested tissues were then triturated with a 25 ml serological pipette (cat #: 75816–090) (VWR, Radnor, PA) to complete digestion. Mammary tissue fragments were then washed in a solution of Hank’s Buffered Saline Solution (HBSS) (cat #: 21-022-CM) (Corning Inc.) containing 2% FBS (R&D Systems) (2% HFBS), then suspended in a solution of FBS (R&D systems) plus 10% dimethyl sulfoxide (DMSO) (cat #: D2650) (Sigma Aldrich), frozen in cryovials at -80°C in isopropanol cryochambers for 24 h, and then stored in liquid nitrogen for long term storage. Prior to use, cryovials were thawed at 37°C for 1–2 min, and mammary tissue fragments were washed in sterile PBS (cat #: 21-040-cv) (Corning Inc.) and kept on ice until xenotransplanted. Tissue fragments of ~200–400 μm in diameter were selected for xenotransplantation and inserted into the cleared mammary fat pad (MFP), as described below, within 0.5 to 3 h after thawing.

### Primary mammary fibroblast culture and injections

Primary early passage (≤ passage, P5) mammary fibroblasts, isolated from cryopreserved mammary tissue fragments and maintained in (DMEM) (Corning Inc.) containing 10% fetal bovine serum (FBS) (R&D Systems) and 1% Pen-Strep (cat #: 30-002-CI) (Corning Inc.) (fibroblast culture media), were cultured for ≤ 14 days prior to being injected into cleared mammary fat pads [38]. For fibroblast isolation, mammary tissue fragments were thawed and resuspended in HBSS (Corning Inc.) containing 2% FBS (R&D Systems) (2% HFBS), then washed in HBSS (Corning Inc.). Fragments were then triturated for 5 min in pre-warmed 0.25% Trypsin-EDTA (cat #: 25-053-CI) (Corning Inc.) and washed in 2% HFBS. To generate single cell suspensions, Dispase II (final conc. 5 U/ml) (cat #: D4693) (Sigma Aldrich) and DNase I (final conc. 0.25 mg/ml) (cat #: LS002138) (Worthington) were added to the solution and fragments were triturated for 5 min. After adding 2% HFBS, single cell suspensions were passed through a 100 μm cell strainer (cat #: 352360) (Corning Inc.), and then a 40 μm cell strainer (cat #: 352340) (Corning Inc.), to remove debris. Cells were counted using a hemacytometer and plated at 3 x 10^6^ cells/well on an adherent 6-well tissue culture plate (cat #: 3516) (Corning Inc.) for 1 h to enrich for adherent fibroblasts [4]. Cells that remained suspended were separated and cultured as mammosphere-derived epithelial cells (MDEC, see below), and adherent fibroblast cultures were trypsinized and re-plated to semi-confluency for injection. After trypsinization, fibroblasts were resuspended at 5 x 10^5^ cells in 50 μl of 1:1 HFH (HBSS (Corning Inc.), 2% FBS (R&D Systems), 0.01M HEPES (cat #: 25-060-CI) (Corning Inc.)):Matrigel Matrix Basement Membrane (8.4 mg/ml protein concentration) (cat #: 354234) (Corning Inc.) [24, 29], and kept on ice until injection into the cleared fat pad. Injections were performed using a 28G needle (cat #: 329424) (BD Pharmingen, Franklin Lakes, NJ). Vehicle injections were performed using a cell-free solution of 50 μl of 1:1 HFH (HBSS (Corning Inc.), 2% FBS (R&D Systems), 0.01M HEPES (Corning Inc.)): Matrigel Matrix Basement Membrane (8.4 mg/ml protein concentration) (Corning Inc.). Following implantations, equine and canine primary mammary fibroblasts were allowed to engraft for 2 weeks prior to analysis and/or mammary tissue fragment xenotransplantation.

### Cleared fat pad surgeries and mating of immunodeficient mice

NOD-scid gamma (NSG) (NOD.Cg-*Prkdc*^*scid*^ *Il2rg*^*tm1Wjl*^/SzJ) mice were purchased from the Cornell Progressive Assessment of Therapeutics (PATh) patient-derived xenograft (PDX) Facility or the Jackson Laboratory (Bar Harbor, ME) and maintained on-site under sterile conditions. All mice were supplied with food (e.g., standard chow) and water *ad libitum*. Fat pad clearing surgeries were performed on 3–4-week-old female mice that were anesthetized with isoflurane (cat #: 200–129) (Dechra Pharmaceuticals, Northwich, UK) mixed with O_2_ (3% vol/vol in induction chamber, then maintained at 2–2.5% vol/vol on nose cone) and received a subcutaneous injection of ketoprofen (3 mg/kg) (cat #: 07-803-7389) (Patterson Veterinary Supply, Loveland, CO), followed by an additional abdominal subcutaneous injection of bupivacaine (5 mg/kg) (National Drug Code #: 0409-1163-18) to reduce post-procedural pain. Timelines for surgical procedures are detailed in S1 Fig. Timeline for baseline surgeries (without additional interventions, i.e., without induced pregnancy or fibroblast injections) is presented in S1a Fig. All surgical procedures were performed on a warming pad within a sterile biosafety cabinet. While under anesthesia, the developing mammary epithelia in the 4^th^ inguinal MFPs were removed to de-epithelialize (clear) the MFPs [21, 24, 29]. Excised portions of the 4^th^ inguinal MFPs were mounted on glass slides and stained by acetocarmine (1.25 mg/ml carmine powder (cat #: C1022) (Sigma Aldrich) in 45% Glacial acetic acid (cat #: A38-212) (Thermo Fisher, Waltham, MA) (see below)) to visualize extracted murine mammary glands and confirm successful clearing. Immediately after clearing, a small incision was made in the remaining fat pad and a donor equine or canine mammary tissue fragment (~200–400 μm in diameter) containing mammary parenchyma and stroma was inserted. Incisions were subsequently sealed with surgical staples (cat #: INS750344) (Cellpoint Scientific, Inc., Gaithersburg, MD) and mice recovered on a warming pad until mobile. Following surgery, mice were given an oral antibiotic suspension of sulfamethoxazole (0.4mg/ml) and trimethoprim (0.08mg/ml) (cat #: 0121-0854-16) (PAI Pharma, Greenville, SC), dissolved in drinking water, for a minimum of 10 days. All mice received additional subcutaneous injections of ketoprofen (3 mg/kg) (Patterson Veterinary Supply) at 24 and 48 h after surgery to alleviate pain. Mice that underwent surgery were housed in cages of ≤ 4 mice and were monitored daily for signs of distress and to ensure proper healing of the abdominal incisions. No signs of distress were observed during the xenograft engraftment period and no challenges were observed (e.g., infection, tumor formation).

For mating procedures, female mice bearing xenografts (6 weeks post-surgery) were paired and placed in mating cages with an adult male NSG mouse. Females were checked for vaginal copulation plugs daily to determine days post-coitus (dpc) and monitored for changes in external appearance to determine gestation progression [39]. For experiments that assessed the mammotropic effects of circulating pregnancy hormones, pregnant mice were euthanized at 18 dpc (S1b Fig). For mice that received primary mammary fibroblast injections, fibroblasts were injected immediately post-clearing, and mammary tissue fragment xenotransplantation surgeries occurred 2 weeks after MFP clearing and fibroblast injections, when mice were aged 5-6-weeks old (S1c Fig). All mice were euthanized by CO_2_ asphyxiation, with CO_2_ flow maintained for 60 s after respiratory arrest, followed by cervical dislocation. Cleared MFPs (4^th^ inguinal MFPs) containing mammary xenografts were then removed and fixed for analysis. All xenografts were assessed ~ 9 weeks after xenotransplantation surgeries, unless otherwise noted.

### Histological staining and immunohistochemistry (IHC)

MFPs containing xenografts were fixed in 10% neutral-buffered formalin (10% NBF) (cat#: HT501128-4L) (Sigma Aldrich) for 24 h at room temperature (RT), stored in 70% EtOH (cat#: 2716GEA) (Decon Labs, Inc., King of Prussia, PA), embedded in paraffin, and sectioned for histological analysis. Picro-Sirius (Sirius) red (cat #: VB-3017) (VitroView, Rockville, MD) and Masson’s Trichrome (MTC) (cat #: HT15-1KT) (Sigma Aldrich) stainings were performed, as per manufacturer’s instructions. Hematoxylin and eosin stainings (H&E) were performed by the Animal Health Diagnostic Center (AHDC), Cornell University College of Veterinary Medicine.

For immunohistochemistry (IHC) analysis of β-lactoglobulin (β-LG), Cytokeratin-14 (CK14), Cytokeratin-18 (CK18), α-Smooth Muscle Actin (α-SMA), and Estrogen receptor-α (ERα) presence, formalin-fixed paraffin embedded (FFPE) tissue sections were deparaffinized in xylene (cat #: 89370–088) (VWR)and rehydrated in EtOH (Decon Labs, Inc.) and H_2_O. Rehydrated tissues were permeabilized in Tris-buffered saline (TBS), containing 0.5% Tween-20 (cat #: J20605-AP) (Sigma Aldrich) when assessing cytoplasmic antigens and in 0.3% Triton X-100 (cat #: J66624) (Thermo Fisher) when assessing nuclear antigens. Antigen retrieval, using a citric acid buffer (10mM citric acid (cat #: C-8532) (Sigma Aldrich), 0.5% Tween-20 (Sigma Aldrich), pH = 6.0), was performed by immersing tissues in 95–100°C buffer for 20 min. Tissues were blocked with a TBS solution containing 1% bovine serum albumin (BSA) (cat #: A3608) (Thermo fisher) and 10% normal goat serum (cat #: 102643–594) (VWR) for 30 min at 37°C. Primary antibodies were diluted in a TBS solution containing 1% BSA (Thermo Fisher) and incubated overnight at 4°C. Endogenous peroxidase activity was blocked by incubating tissues in 0.3% H_2_O_2_ (cat #: H325-500) (Thermo Fisher) for 15 min at RT. Secondary antibodies were diluted as described above (TBS containing 1% BSA (Thermo Fisher)), and incubated for 1 h at RT. Antibody information and dilution concentrations are detailed in S1 Table. To improve immunolabelling signal for CK14 and CK18, tissues were incubated with anti-mouse IgG (H+L), biotin-SP (Jackson, West Grove, PA) for 1 h at RT, washed, and then incubated with Streptavidin, Horseradish peroxidase (HRP)-conjugated (Sigma Aldrich) for 1 h at RT. Colorimetric signals were developed with 3-Amino-9-Ethylcarbazole (AEC) substrate kit (cat #: 551015) (BD Pharmingen) and tissues were counterstained with Gill’s hematoxylin (cat #: 6765007) (Thermo fisher). Isotype control antibodies (S1 Table) were incubated with tissue serial sections at identical concentrations as corresponding primary antibodies to ensure that immunofluorescent (IF) and IHC signals were not due to non-specific binding. IHC labeling of vimentin and Marker of proliferation Ki-67 (Ki-67) was performed by Cornell AHDC. Briefly, FFPE tissue sections were processed using a Bond Max automated IHC stainer (Leica Biosystems, Nussloch, Germany). Ki-67 signal was developed using the BOND Polymer Refine Red Detection system (cat #: D59390) (Leica Biosystems), and vimentin signal was developed using the BOND Polymer Refine Detection system (cat#: D59800) (Leica Biosystems). Diluent-only (Bond Primary Antibody Diluent) (cat #: AR9352) (Leica Biosystems) controls (no 1° ctrl) were performed alongside Ki-67 and vimentin IHC analyses to ensure signals were not derived from non-specific binding. IHC labelled tissue sections were mounted using Glycergel Aqueous Mounting Medium (cat #: C0563) (Dako, Glostrup, Denmark).

For IF labeling, the protocol was followed as described above. Fluorescent signal was developed using an Alexa Fluor-488 conjugated antibody (S1 Table), counterstained with DAPI dilactate (4′,6-diamidino-2-phenylindole) (1:20000) (cat #: 76482–848) (AAT Bioquest, Inc, Pleasanton, CA), and tissues were imaged using an Olympus Fluoview FV3000 confocal microscope (Olympus, Tokyo, Japan) and processed using ImageJ software (1.54f) (Java 1.8.0_172 (64-bit)) (https://imagej.nih.gov/ij/). To quantify fluorescence intensity, corrected total cell fluorescence (CTCF) was performed. IF images were converted to grayscale and individual xenograft mammary ductal structures were outlined using ImageJ software to determine structure area and integrated density (IntDen). Background mean fluorescence was determined by measuring mean fluorescence within non-fluorescent regions of each tissue section. CTCF = IntDen–(Structure area*Mean fluorescence of background) [40, 41]. CTCF for each whole xenograft was determined by averaging the CTCF of each xenograft ductal structure within each tissue section.

### Clear unobstructed brain/body imaging cocktails (CUBIC) tissue clearing and light sheet imaging

CUBIC-based tissue clearing was performed as described [42]. Briefly, MFPs containing xenografts were fixed in 4% paraformaldehyde (PFA) (cat #: J19943-K2) (Thermo Fisher) for 24 h, washed in PBS (Corning Inc) (3 x 1 h) and placed in CUBIC Reagent 1A (N,N,N’,N’-tetra-kis(2-hydroxypropyl)-ethylenediamine (5% (w/w)) (cat#: T0781) (TCI, Tokyo, Japan), triton-X100 (10% (w/w)) (Thermo Fisher), urea (10% (w/w)) (cat #: BP169-500) (Thermo fisher), and NaCl (25 mM) (cat#: S271) (Thermo fisher) in distilled water) on a rotator for 5 days at 37°C, with solution changed every 24–48 h. For IF labeling, tissues were washed in PBS (Corning Inc) then blocked in blocking solution (triton-X100 (0.5% (w/v) (Thermo Fisher), 10% normal goat serum (v/v) (VWR)) overnight at 4°C. Tissues were then immersed in Alexa Fluor^™^ 488 Phalloidin (Invitrogen, Waltham, MA) diluted in blocking solution (1:500) for 5 days at 4°C. Tissues were then washed in PBS (Corning Inc) and immersed in CUBIC Reagent 2 (sucrose (44% (w/w)) (cat#: S5-500) (Thermo fisher), urea (22% (w/w)) (Thermo Fisher), triethanolamine (9% (w/w)) (cat #:90279-100ML) (Sigma Aldrich), triton-X100 (0.1% (w/w) (Thermo Fisher)) in distilled water) for 2 days at 37°C. To finish clearing and in preparation for light sheet imaging, MFPs were embedded in Ultrapure Agarose (1.5% (w/v)) (cat #:16500–500) (Invitrogen) in CUBIC Reagent 2 within polymethylmethacrylate square spectrophotometer cuvettes (cat #: 111157) (Globe Scientific, Mawah, NJ). Fluorescent images were taken using a Light Sheet microscope (LaVision Biotech, Bielefeld, Germany) designed around an Olympus MVX-10 zoom macroscope (Olympus). Xenograft tissues were imaged using wavelengths at 488 nm (Alexa Fluor^™^ 488 Phalloidin) and 561 nm (autofluorescence) and 3D renderings and z-stack (10 μm optical thickness) videos were generated using Arivis Vision 4D software (version 3.4) (Zeiss, Dublin, CA). Structural boundaries and morphology of mammary xenografts were defined in Arivis Vision 4D software (version 3.4) (Zeiss) by using anatomical mammary tissue landmarks (e.g., mammary lumina, bilayered mammary epithelia). Xenograft vascularization was assessed by differences in autofluorescence intensity and structural morphology, and manually outlined.

### Nucleic acid extraction and quantitative RT-qPCR analysis

To validate the presence of equine or canine genomic DNA (gDNA), DNA was extracted from FFPE tissue scrolls containing cleared MFPs bearing mammary xenografts. Paraffin was removed using xylene (cat#: 9460–11) (VWR) and EtOH (cat #: E7023-500ML) (Sigma Aldrich) and DNA was extracted using the DNeasy Blood and Tissue kit (cat #: 69506) (Qiagen, Hilden, Germany), as per manufacturer’s instructions. PCR amplicons were generated using species-specific primers for secretory carrier membrane protein 3 (*Scamp-3*) (murine-specific) and beta-2-microglobulin (*B2M)* (either equine- or canine-specific) and analyzed on 2% agarose gels. For real-time quantitative (RT-q) PCR, RNA was extracted from paraffin scrolls using the FFPE RNA extraction kit (cat #: 73504) (Qiagen), as per manufacturer’s instructions. cDNA was synthesized using M-MLV Reverse Transciptase (cat #: PR-M170A) (Thermo fisher) and RT-qPCR reactions were performed using SYBR^®^ Green Supermix (cat #: 172–5124) (Bio-Rad, Hercules, CA). All RT-qPCR reactions were performed using a QuantStudio 3 Real Time PCR system (Thermo fisher). Target gene expression was normalized to *GAPDH* expression and fold change (FC) was determined using the 2^-ΔΔCt^ method then transformed using the Log_2_(FC) function. Undetected samples were analyzed as Cycle threshold (Ct) = 40. Primers used for gDNA PCR and RT-qPCR are listed in S2 Table.

### Acetocarmine staining of wholemount tissues

MFPs containing extracted mammary epithelia and de-epithelialized (cleared) MFPs bearing xenografts were excised, spread evenly on a glass slide (cat #: 48311–703) (VWR), and air-dried for 5 min. Tissues were fixed in 10% NBF (Sigma Aldrich) for 24–48 h and stored in 70% EtOH (Decon Labs, Inc.). For staining, tissues were first rinsed in H_2_O for 15 min, dehydrated in 1 h changes of 95% and 100% EtOH (Decon Labs, Inc.), and cleared in xylene (cat #: 89370–088) (VWR) for 2 h, then submerged in acetocarmine solution (1.25 mg/ml carmine powder (Sigma Aldrich) in 45% Glacial acetic acid (Thermo Fisher)) for 48 h. After staining, tissues were rinsed, dehydrated, and cleared as described above, and mounted using Permount mounting media (cat #: 022–208) (Thermo fisher). When assessing the presence of blebbing structures on xenografts, all acetocarmine stained tissues were assessed by an observer blinded to each condition. Wholemounted xenograft surface area (mm^2^) was measured using ImageJ software (1.54f) (Java 1.8.0_172 (64-bit)) (https://imagej.nih.gov/ij/). Briefly, the ImageJ scale feature was calibrated to a defined distance using a scale micrometer, then the xenograft structural boundaries were traced by an observer blinded to each condition at equal magnifications to measure surface area (mm^2^).

### Mammosphere-derived epithelial cell (MDEC) culture, fibroblast conditioned media generation (CM), and BrdU proliferation assay

Mammosphere-derived epithelial cells (MDEC) populations were generated as previously described [4, 37, 43] from mammary tissue fragments used in this study. Briefly, mammary tissues and single cell suspensions were isolated as described above and 3 x 10^6^ cells were plated in adherent 6-well culture plates (Corning Inc.). Primary cells incubated for 1 h over two cycles, and non-adherent cells, which were depleted of mammary fibroblasts, were collected and plated in suspension to form mammospheres [43]. Mammospheres were then seeded on adherent T75 flasks (cat #: 07-000-228) (Thermo Fisher), incubated at 37°C and 5% CO2, in media containing DMEM/F12 (50/50) (Corning Inc.) with 10% FBS (R&D Systems), 2% B27 (cat #: 12587–010) (Thermo Fisher), 1% antibiotic/antimycotic (cat #: 30-004-CI) (Invitrogen), 10 ng/mL basic fibroblast growth factor (cat #: SRP4037) (BioVision, Milpitas, CA), and 10 ng/mL epidermal growth factor (cat #: E9644) (Sigma Aldrich) (EpSC medium) to generate MDEC cultures.

To generate primary mammary fibroblast conditioned media (CM), early passage (≤ P5) equine or canine fibroblasts were seeded at 1.33 x 10^4^ cells/cm^2^ in T25 culture flasks (cat #: 07-000-226) (Thermo Fisher) and incubated overnight at 37°C in 5% CO2 in fibroblast culture media. Fibroblast culture media was removed, then cell monolayers were washed twice in sterile 1x PBS (Corning Inc). Four ml of serum-free (sf) DMEM (Corning Inc.) was added to fibroblast cell monolayers and incubated for 24 h to generate fibroblast CM. CM was removed and centrifuged twice at 300 x g for 10 min, with supernatants moved to a sterile tube after each spin to remove cell debris before proceeding to BrdU proliferation analysis.

BrdU proliferation analysis was performed using the BrdU Cell Proliferation ELISA kit (colorimetric) (cat #: ab126556) (Abcam, Cambridge, UK), as per manufacturer’s instructions. Briefly, biological replicates (n = 3) of equine and canine MDEC cultures were plated in triplicate at 5 x 10^3^ cells/well in a 96-well cell culture plate (cat #: 3596) (Corning Inc.) and allowed to recover for 24 h. EpSC media was removed and fibroblast CM containing 1X BrdU reagent was added, after which cells were incubated for 24 h. Cells were then washed, fixed, and BrdU was labeled, as per manufacturer’s instructions. BrdU incorporation was assessed via spectrophotometric analysis using a Tecan Infinite M200 Pro Microplate Reader (Tecan, Männedorf, Switzerland). Colorimetric reads were taken at 450 nm and background reads (550 nm reads, 450nm reads from “no BrdU” control wells) were subtracted prior to statistical analysis.

### Statistical analysis

Student’s two-tailed t-tests were used to determine statistical significance unless otherwise noted. Paired two-tailed t-tests were performed when assessing matched samples for BrdU proliferation analysis. An alpha level of 0.05 was used for all tests. (* P < .05, ** P < .01, *** P < .001, and **** P < .0001). All above statistical analyses were performed using GraphPad Prism (version 10.1.2 (324)) (GraphPad Software, La Jolla, CA).

## Results

### Xenotransplanted mammary tissue fragments from equine and canine donors engraft in murine mammary fat pads and establish mammary xenografts

**Fig 1:**
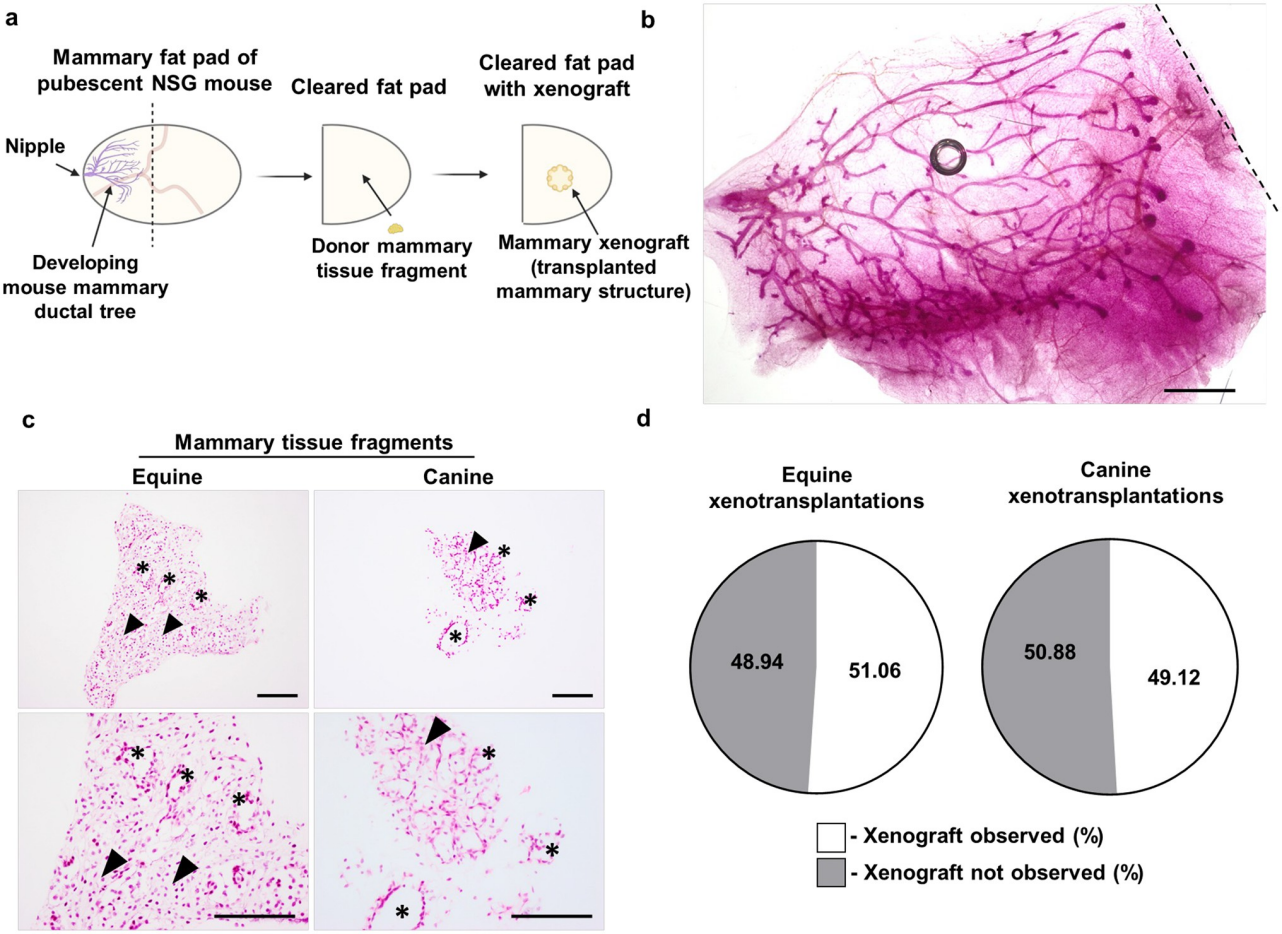
Overview of mammary fat pad clearing and xenotransplantation procedure. **(a).** Diagram depicting the mammary fat pad (MFP) clearing and xenotransplantation approach. **(b).** Acetocarmine-stained pubescent murine mammary gland extracted from the 4^th^ MFP of a 3-week-old NOD scid gamma (NSG) mouse. Dotted line indicates location of incision that separated the developing mammary gland from the remaining fat pad. Scale bar = 500 μm. **(c).** Representative mammary tissue fragments from equine and canine donors stained with H&E. Arrowheads indicate regions of fibrous supportive stroma and asterisks (*) indicate representative examples of mammary ductal structures. Scale bar = 100 μm. **(d).** Pie chart of percent (%) engraftment success rate of xenotransplantations, as determined by histological analysis. Counts determining engraftment success rate per experiment are presented in S3 Table.

Developing mammary glands were extracted from pubescent (3–4 weeks old) immunodeficient NSG mice to de-epithelialize (clear) the mammary fat pads (MFPs) [21, 24, 29], and mammary tissue fragments from equine or canine donors were bilaterally xenotransplanted into the residual stroma (Fig 1a. Successful clearing of the MFP was confirmed by acetocarmine staining of the removed murine endogenous mammary ductal tree (Fig 1b). Mammary tissue fragments from both species ranged from ~ 200–400 μm in diameter and contained both parenchymal and stromal components, as shown by mammary ducts with characteristic mammary lumina surrounded by a fibrous supportive stroma (Fig 1c). Nine weeks after the xenotransplantation surgery (S1a Fig), MFPs containing xenografts were extracted for downstream analyses. Based on the presence or absence of mammary xenografts within formalin-fixed paraffin-embedded (FFPE) serial sections and acetocarmine stained wholemounts, we report a ~ 50% engraftment success rate (Fig 1d).

**Fig 2:**
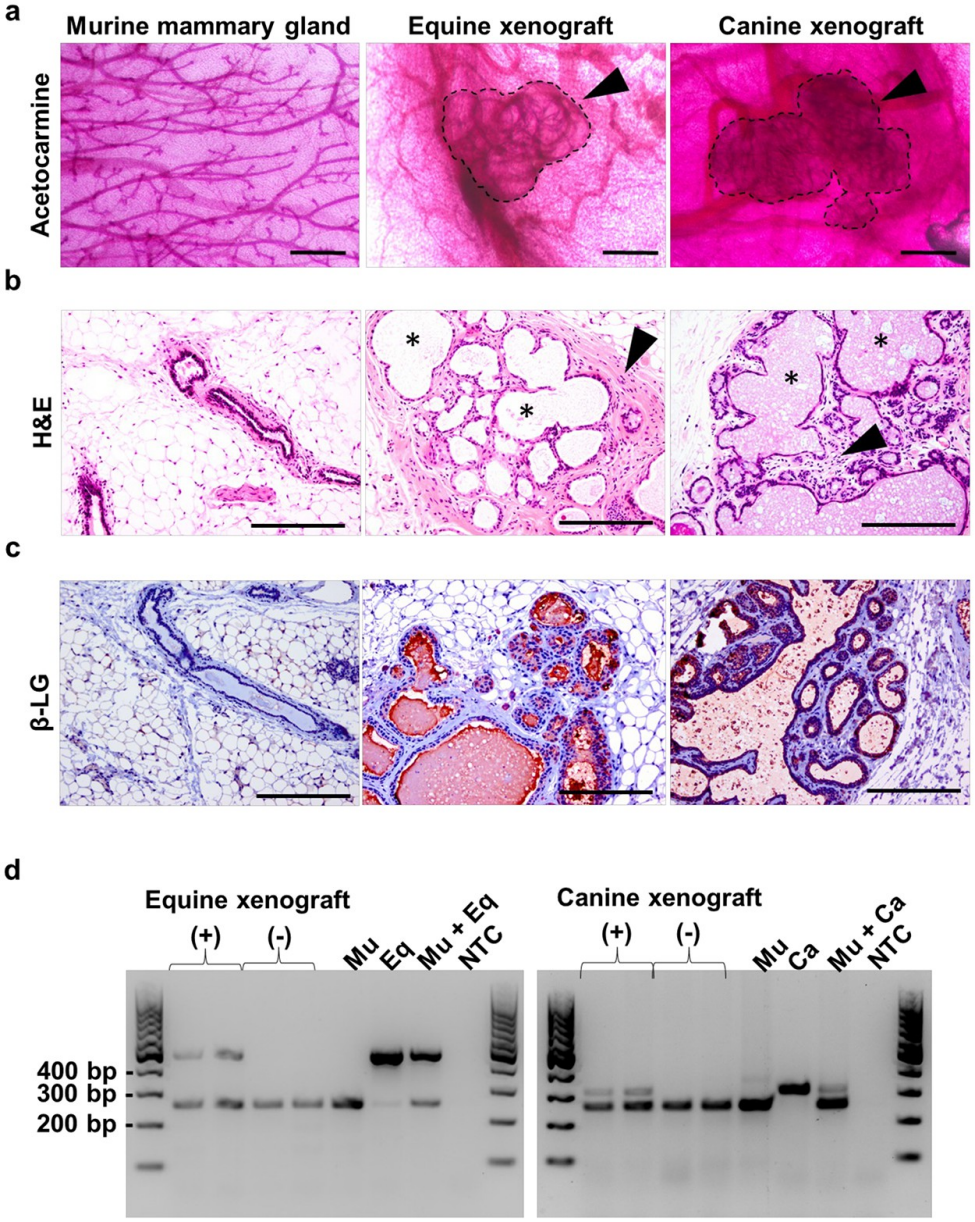
Histological analysis and species identity confirmation of equine and canine mammary xenografts. **(a).** Acetocarmine-stained murine mammary gland and equine and canine mammary xenografts within the cleared MFPs. Arrowheads indicate xenografts and dotted black lines designate xenograft borders. Scale bar = 500 μm **(b).** H&E staining of tissue sections containing murine mammary gland and mammary xenografts. Arrowheads indicate xenograft stroma and asterisks (*) indicate representative examples of xenograft lumina. Scale bar = 500 μm. **(c).** IHC analysis of β-lactoglobulin (β-LG) labeling in murine mammary gland and mammary xenografts. Scale bar = 500 μm. **(d).** PCR verification of xenograft species identity using species-specific primers (Murine (Mu): *Scamp3*, Equine (Eq): *B2M*, Canine (Ca): *B2M*). Genomic DNA extracted from FFPE sections containing (+) or lacking (-) mammary xenografts and from respective donor mammary primary cells as positive control. NTC = “no template” control. Raw gel images are presented in S3 Fig.

Mammary xenografts from both equine and canine mammary donors were observed in cleared MFPs (Fig 2). Wholemount staining showed that equine and canine xenografts resemble mammary lobules containing multiple clustered acini that are structurally distinct from the branching mammary ductal tree characteristic of the mouse mammary gland (Fig 2a). These regionally-confined structures lack detectable ductal branching and resemble mammary xenografts derived from other mammalian donors such as human [29] and cow [15, 24, 27]. Hematoxylin and eosin (H&E) staining showed that both equine and canine xenografts are comprised of multiple acinar-like structures containing hollow lumina that are surrounded by a layer of supportive stroma (Fig 2b). To confirm the correct species of origin and to assess the presence of secretory luminal cells in the mammary xenografts, immunohistochemistry (IHC) was performed to detect β-lactoglobulin (β-LG), a whey protein secreted in the milk of ruminants, horses, and dogs, but absent in humans and rodents [44, 45]. The presence of β-LG in luminal cells and within lumina verified that these structures were derived from equine or canine donor tissues (Fig 2c). Importantly, the mammary xenografts showed structural (S2a Fig) and β-LG presence (S2b(i) Fig) similarities when compared to donor species glands. To further validate tissue engraftment and species identity, genomic DNA (gDNA) was extracted from paraffin scrolls of MFPs that were confirmed by H&E analysis to contain or not contain mammary xenografts. PCR analysis using species-specific gDNA primers confirmed the presence of equine or canine gDNA in sections containing xenografts, but not in those without xenografts (Fig 2d). As expected, PCR reactions using murine-specific *Scamp3* primers resulted in amplicons (246 bp) in all samples containing mouse gDNA, and equine- and canine-specific *B2M* primers resulted in amplicons (equine *B2M*: 442 bp; canine *B2M*: 303 bp) in MFPs containing xenografted tissues and the equine or canine positive controls (Fig 2d).

### Mammary xenografts proliferate and display parenchymal and stromal characteristics similar to donor equine and canine mammary glands

**Fig 3:**
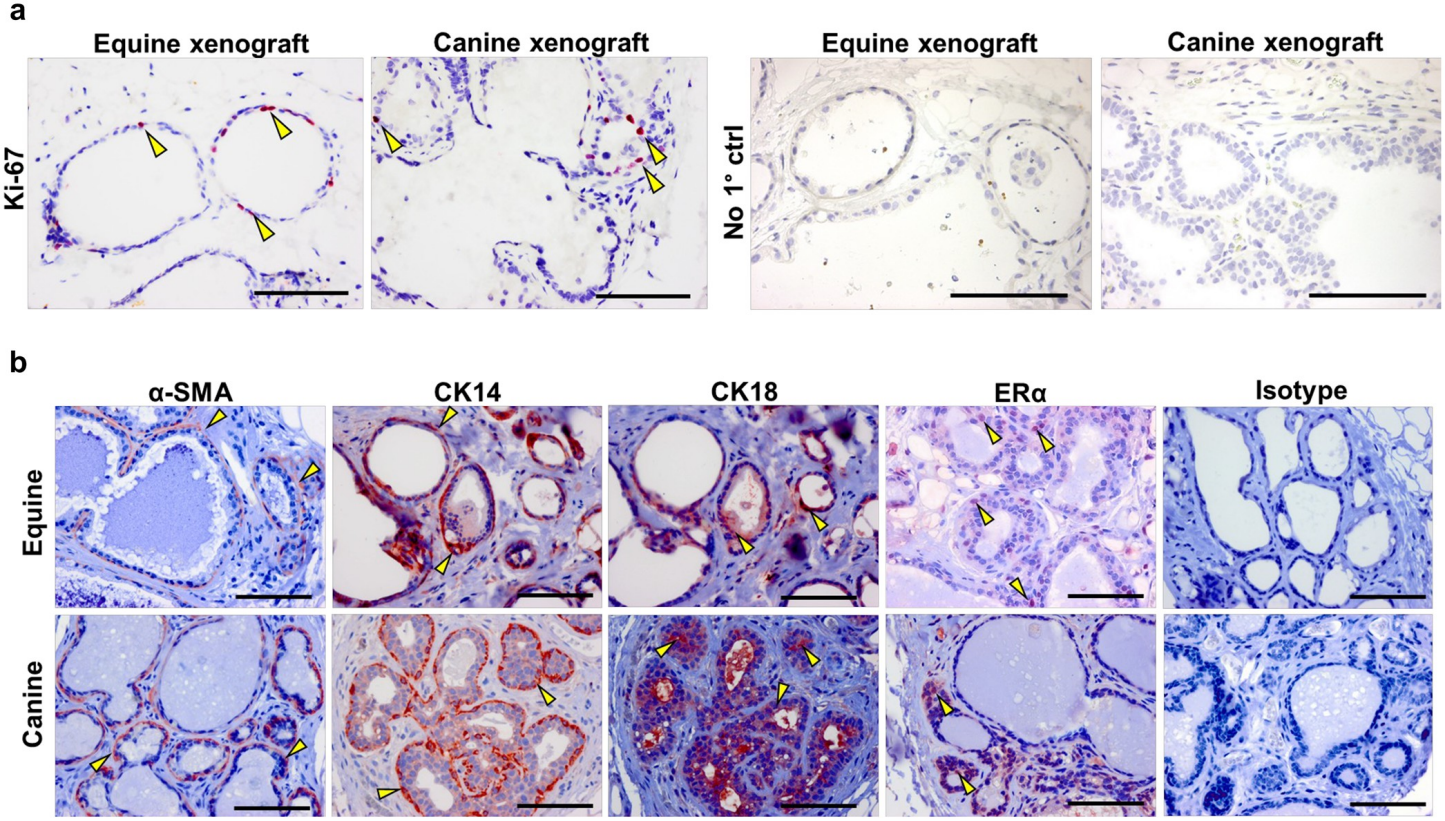
Mammary cell proliferation and parenchymal characteristics. **(a)**. IHC analysis of Ki-67 presence in equine and canine xenografts. No primary antibody control tissues (No 1° ctrl) are presented. **(b).** IHC analysis of α-smooth muscle actin (α-SMA), cytokeratin-14 (CK14), cytokeratin-18 (CK18) and estrogen receptor-α (ERα) labeling in equine and canine mammary xenografts. Representative tissues treated with isotype controls are presented. Arrowheads indicate positive IHC labeling (red colorimetric indicator) Scale bar = 100 μm.

Equine and canine mammary xenografts were characterized by IHC using functional and structural markers (Fig 3). The proliferation marker Ki-67 (Ki-67) was used to assess proliferation of the engrafted mammary tissue fragments, indicating survival within the host MFP. Ki-67^+^ cells were observed in both equine and canine mammary xenografts with presence localized primarily to the inner luminal cell layer (Fig 3a). To determine if the mammary xenografts recapitulate the structural architecture and protein labeling patterns observed in donor glands, markers for myoepithelial cells (i.e., α-smooth muscle actin (α-SMA) and cytokeratin-14 (CK14)) and luminal cells (i.e., cytokeratin-18 (CK18)) were evaluated (Fig 3b). Similar to what is observed in equine and canine mammary glands [31, 32] (S2b(ii) Fig), α-SMA and CK14 labeling was restricted to the outer myoepithelial cell layer and CK18 labeling was restricted to the inner luminal cells of the xenografts, indicating a bilayered architecture (Fig 3b). Presence of estrogen receptor-alpha (ERα) was also assessed, as mammary gland responsiveness to mammotropic hormones (e.g., estrogens) is a defining feature of mammary gland growth and functionality [46]. As expected, we observed ERα presence in the nuclei of luminal cells of both equine and canine xenografts (Fig 3b), mirroring characteristics of the donor glands [31, 32] (S2b(ii) Fig) and indicating that these mammary xenografts may be responsive to hormonal stimulation.

**Fig 4:**
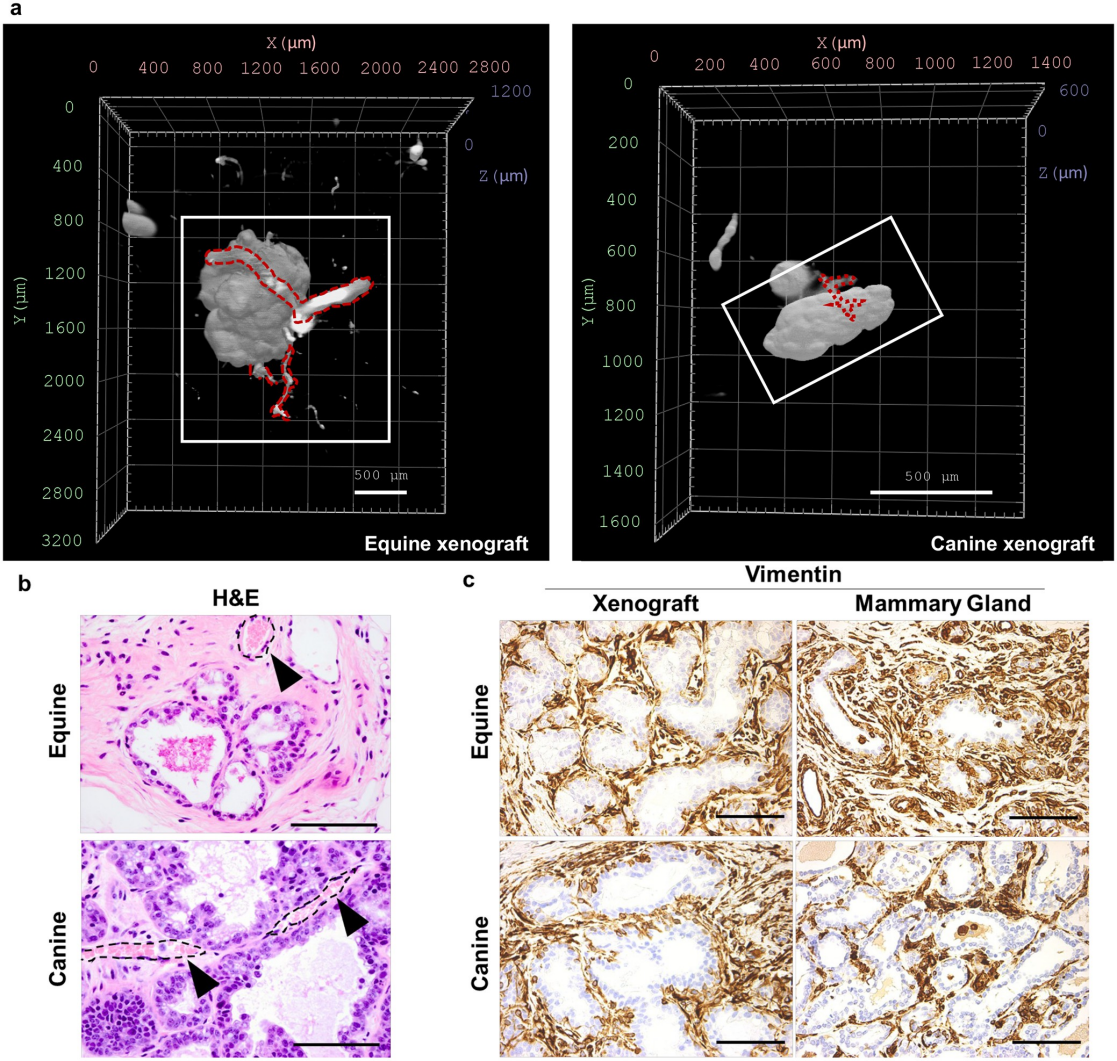
Xenograft structure, vascularization, and stromal characteristics. **(a).** Volume renderings of equine and canine mammary xenografts generated following wholemount light sheet imaging. Images captured with autofluorescent signal (561 nm). White box denotes mammary xenograft and dotted red lines highlight surrounding vasculature. Scale bars = 500 μm. **(b).** H&E analysis of equine and canine mammary xenografts. Dotted lines and arrowheads indicate blood vessels. **(c).** IHC analysis of vimentin presence in equine and canine mammary xenografts and donor mammary tissues. Scale bars = 100 μm.

To visualize mammary xenografts in a three-dimensional space and assess vascularization, MFPs containing mammary xenografts were rendered optically transparent using a Clear Unobstructed Brain Imaging Cocktails (CUBIC)-based approach and imaged using light sheet microscopy [42]. In concordance with observations of acetocarmine- and H&E-stained xenograft tissues using 2D imaging techniques (Fig 2a and 2b), 3D imaging also showed that equine and canine xenografts consist of multiple acini confined to the xenotransplantation site (Fig 4a, S1–S3 Videos). Importantly, autofluorescent imaging, which is sufficient to define anatomical boundaries within tissues [47, 48], revealed blood vessels surrounding and penetrating equine and canine xenografts, indicating endothelial cell recruitment (Fig 4a, S1–S3 Videos). The extensive vasculature within the xenograft stroma of both species was corroborated by H&E staining (Fig 4b). Performing IHC analysis to evaluate vimentin presence, a filamentary protein present in both myoepithelial cells [49] and stromal fibroblasts [31, 32], showed that the mammary parenchyma of each xenograft was surrounded by an extensive fibroblast-rich stroma (Fig 4c), further demonstrating structural similarity to what is observed in donor mammary glands. Important to note is that: (i) the antibody used for vimentin labeling is non-reactive in mouse [50], as indicated by a lack of vimentin labeling in vimentin-rich murine tissues (i.e., skin and mammary gland [51–54]) following IHC (S4 Fig), and (ii) positive labeling was localized solely to the xenotransplantation site and absent in other extracellular matrix (ECM)-rich, fibrous regions of the MFP, suggesting that the observed stroma of equine and canine xenografts is largely derived from the stromal cells of the xenotransplanted tissue fragments.

### Host pregnancy upregulates expression of milk-associated genes and alters mammary xenograft proliferation

**Fig 5:**
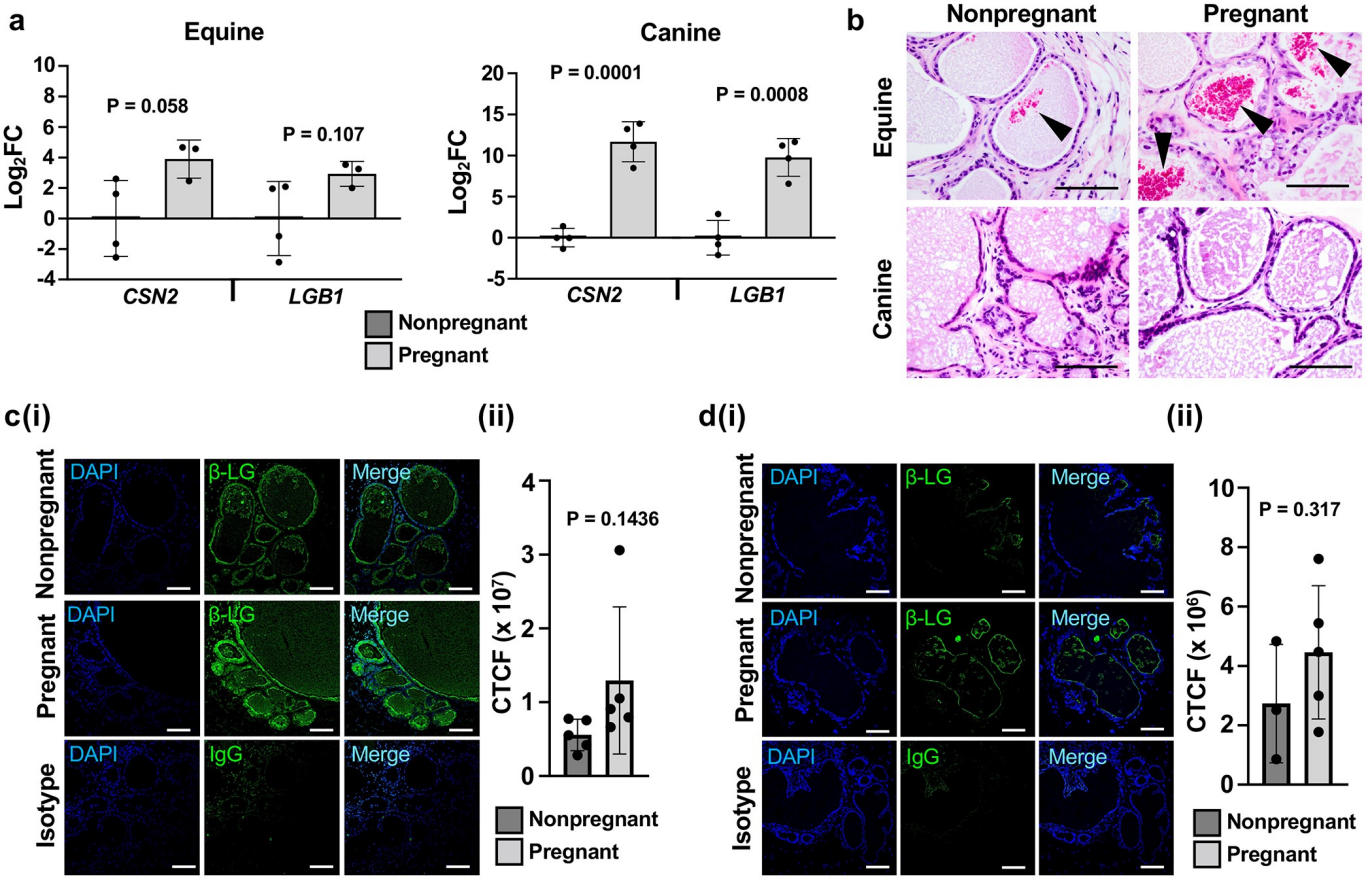
Mammary xenografts respond to endogenous mammotropic hormone stimulation. **(a).** RT-qPCR analyses of lactation-associated genes, β-casein (*CSN2*) and β-lactoglobulin (*LGB1*), MFPs containing equine or canine xenografts from nonpregnant or pregnant host mice. Fold change (FC) determined via the 2^-ΔΔCt^ analysis method and transformed via the Log_2_(FC) function. Error bars represent standard deviation (SD). Statistical significance was assessed using unpaired (Student’s) t-test (P ≤ 0.05). **(b).** H&E analysis of xenografts in nonpregnant or pregnant host mice. Arrowheadss indicate thickened eosinophilic deposits within equine xenografts. Scale bar = 100 μm. **(c).** Immunofluorescent (IF) labelling of β-lactoglobulin (β-LG) in equine xenografts from nonpregnant and pregnant mice **(i)** and corrected total cell fluorescence (CTCF) intensity quantitation **(ii). (d).** IF labelling of canine xenografts from nonpregnant and pregnant mice **(i)** and CTCF intensity quantitation **(ii).** Immunoglobulin G (IgG) antibody was used as isotype control on xenografts from pregnant mice. Tissues counterstained with DAPI (4′,6-diamidino-2-phenylindole). Scale bar = 100 μm. Error bars represent standard deviation (SD). Statistical significance was assessed using unpaired (Student’s) t-test (P ≤ 0.05).

Pregnancy promotes the production of circulating ovarian mammotropic hormones and growth factors that drive mammary development [55, 56]. It is well-established that many of these bioactive factors are species cross-reactive [29, 57–59]. To validate hormone responsiveness, and thus, functionality of the mammary xenografts, host mice were mated and xenografts were assessed 18 days post-coitus (dpc) (S1b Fig), a period prior to parturition during which circulating levels of mammotropic ovarian hormones are high and the murine mammary gland has developed to facilitate lactation [29, 60, 61] (S5 Fig). Gross anatomical changes in mated mice at 18 dpc are shown in S5a Fig, and representative developmental changes of the murine mammary gland within the 3^rd^ inguinal mammary fat pad are presented (S5b Fig) to indicate typical murine mammary development at this timepoint. Changes in expression of the milk protein-associated genes β-casein (*CSN2*) [9, 46] and β-lactoglobulin (*LGB1*) [9] were assessed by RT-qPCR analyses of MFPs containing xenografts using equine or canine-specific primers. Equine and canine xenografts recovered from pregnant hosts showed an upregulation in both *CSN2* and *LGB1*, albeit this did not reach significance for the equine xenografts due to a high variation in gene expression in the nonpregnant group (Fig 5a). H&E imaging showed the presence of thickened eosinophilic deposits, suggestive of lipid synthesis and proteinaceous fluid production [29, 56] within the lumina of equine, but not canine, xenografts (Fig 5b), with slightly greater abundance of deposits observed in the equine xenografts from pregnant hosts. Despite a lack of dense thickened deposits, eosinophilic secretions were still present within the lumina of the canine xenografts (Fig 5b). IF imaging of β-LG in nonpregnant and pregnant equine (Fig 5c(i)) and canine (Fig 5d(i)) xenografts revealed a baseline level of β-LG presence within the lumina regardless of host reproductive state. Quantification of fluorescence within the mammary xenograft lumina using a corrected total cell fluorescence (CTCF) approach revealed increased presence of β-LG labelling within both equine (Fig 5c(ii)) and canine (Fig 5d(ii)) xenografts from pregnant hosts, mirroring the expression trends of the RT-qPCR analyses (Fig 5a). Notably, significance in fluorescence intensity between nonpregnant and pregnant equine and canine xenografts did not reach statistical significance due to intra-group variations in CTCF (Fig 5c(ii) & 5d(ii). Regardless of the minor responses of the equine xenografts to host pregnancy, these data indicate functional mammary differentiation and lactation of xenografts when exposed to increased concentrations of host-derived circulating hormones via pregnancy.

**Fig 6:**
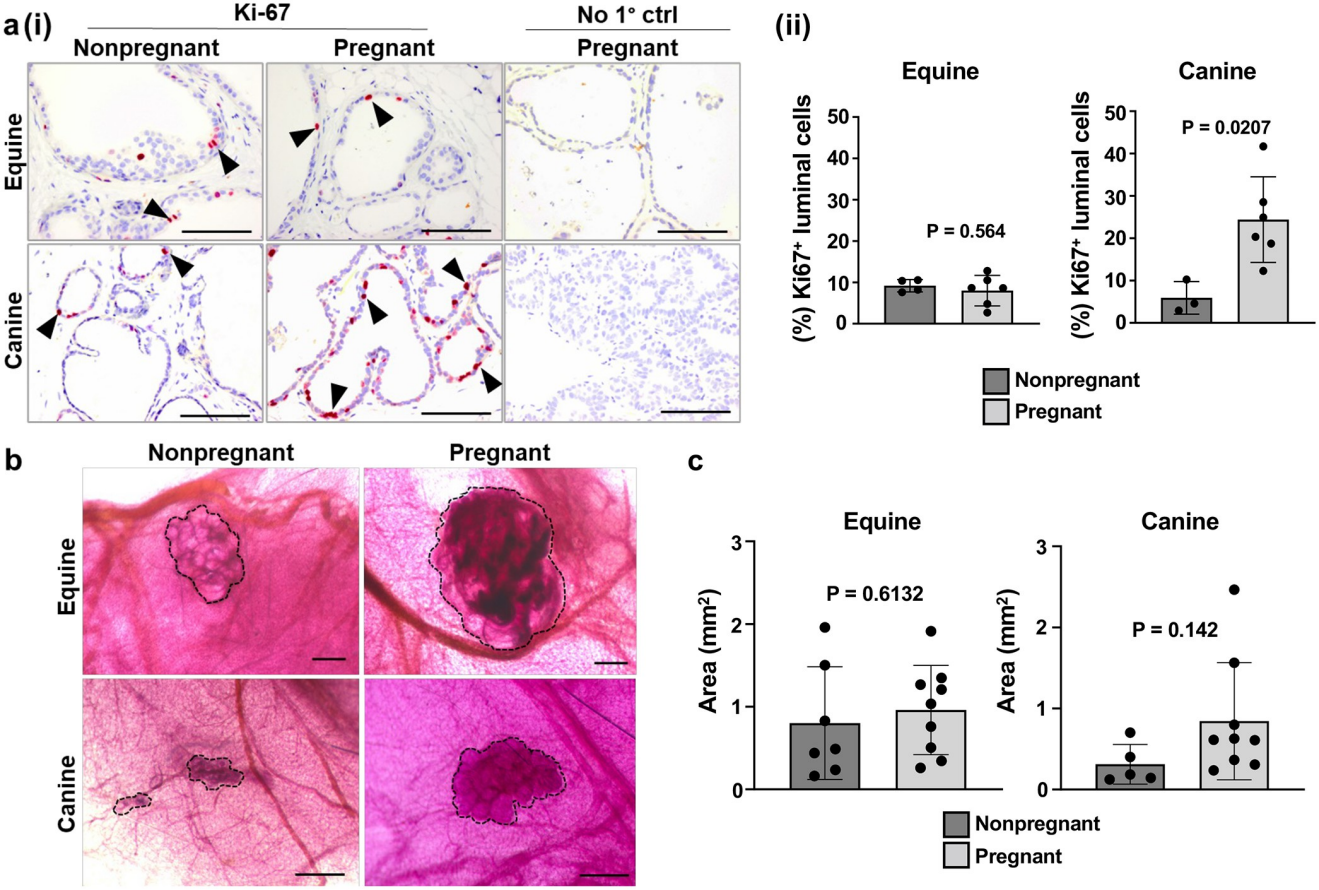
Xenograft whole mounts and size analysis of xenografts in response to endogenous mammotropic hormone stimulation. **(a).** IHC analysis of Ki-67 positivity in mammary xenografts derived from nonpregnant or pregnant host mice. Arrowheads indicate representative Ki-67^+^ cells. Scale bar = 100 μm. **(i)**. Quantification (%) of Ki-67^+^ mammary luminal cells. **(ii)**. Error bars represent standard deviation (SD). Statistical significance was assessed using unpaired (Student’s) t-test (P ≤ 0.05). **(b).** Acetocarmine-stained mammary xenografts from nonpregnant and pregnant host mice. Dotted black lines designate xenograft borders. Scale bar = 500 μm. **(c).** Surface area (mm^2^) quantifications of equine **(i)** and canine **(ii)** mammary xenografts. Error bars represent standard deviation (SD). Statistical significance was assessed using unpaired (Student’s) t-test (P ≤ 0.05).

It is well-established that in addition to lactogenesis, pregnancy also drives increased cell number and glandular surface area [62]. A characteristic of this process is increased proliferative index of epithelial cells as the gland develops to facilitate lactation [63–65]. To that end, IHC labelling of Ki-67 in nonpregnant and pregnant equine and canine xenografts (Fig 6a(i)) showed a significant increase in the percentage of Ki-67^+^ mammary luminal cells was observed in canine, but not equine, xenografts recovered from pregnant mice (Fig 6a(ii)). To assess xenograft structural alterations, wholemounts containing equine and canine mammary xenografts from nonpregnant and pregnant mice were stained with acetocarmine, which showed a lobule-like, spherical morphology confined to the xenotransplantation site irrespective of donor species or host pregnancy status (Fig 6b). To determine if host pregnancy drives growth, xenograft surface area was assessed [28]. A trend towards increased average xenograft surface area following pregnancy was observed, especially in the canine xenografts; however, sizes were highly variable within groups and thus, statistical significance was not reached (Fig 6c). Although the reasons for this remain elusive, the varied xenograft responses could be due to horses, dogs, and mice displaying *in vivo* differences in circulating hormone concentrations during pregnancy [66–68], or due to differences in mammary developmental stages between equine and canine xenografts in response to ovarian hormones, which would not be captured in these experiments as all xenografts were recovered and analyzed at the same time point; 18 dpc. Collectively, and despite some discrepancies between both mammals, these data demonstrate that mammary xenografts responded to hormonal stimulation, indicating xenograft functionality.

### Preimplantation of mammary fibroblasts does not promote mammary ductal branching in equine and canine xenografts

**Fig 7:**
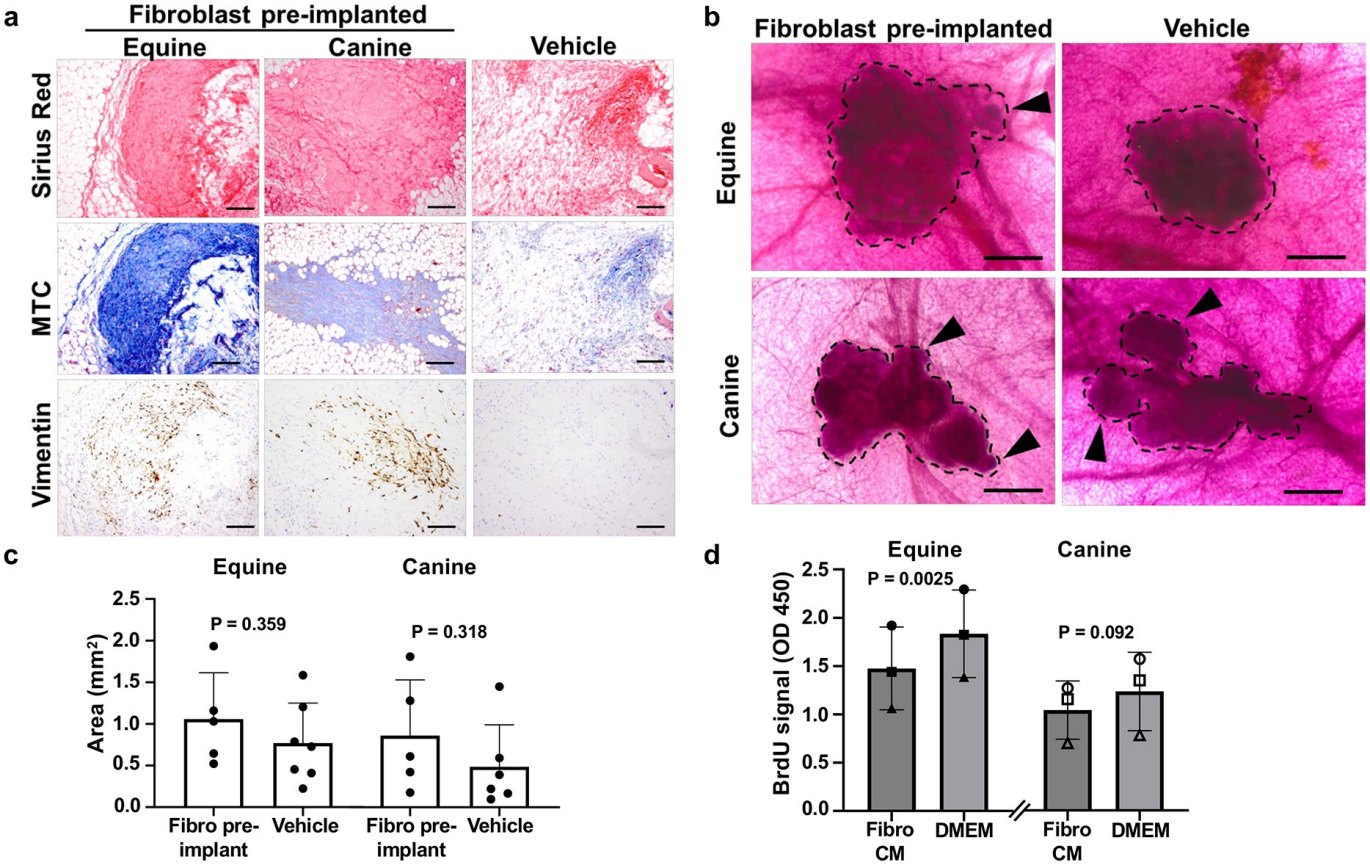
Pre-implantation of mammary fibroblasts and assessment of ductal morphogenesis. **(a).** Sirius red and Masson’s Trichrome (MTC) histological stains, and IHC analysis of vimentin presence in vehicle-injected and mammary fibroblast pre-implanted MFPs (2 weeks post-injection). Scale bar = 200 μm. **(b).** Acetocarmine-stained mammary xenografts from vehicle-injected and mammary fibroblast pre-implanted MFPs. Black dotted lines designate xenograft borders. Arrowheads indicate regions of xenograft blebbing. Scale bar = 500 μm. **(c).** Surface area (mm^2^) quantifications of acetocarmine stained mammary xenografts within vehicle-injected or fibroblast pre-implanted MFPs. Error bars represent standard deviation (SD). Statistical significance was assessed using unpaired (Student’s) t-test (P ≤ 0.05). **(d).** BrdU assay assessing equine and canine MDEC proliferation over 24 h treatment with species-matched mammary fibroblast CM compared to control base media (DMEM). Symbols (circle, square, and triangle) represent individual MDEC cultures of both species. Error bars represent standard deviation (SD). Statistical significance was assessed using paired two-tailed t-tests (P ≤ 0.05).

While mammary lobule-like structures are commonly observed following equine and canine mammary cell xenotransplantation, indicated by xenografts consisting of clustered acini (Fig 2a and 2b, S1–S3 Videos), ductal branching into the MFP is typically absent [24, 29]. Previous reports using human mammary epithelial cells showed that pre-implantation of species-matched fibroblasts within the MFP were capable of driving xenograft ductal morphogenesis [29, 38]. This is based on the knowledge that soluble ligands produced by stromal fibroblasts, such as fibroblast growth factor (FGF), fibroblast-derived ECM components, such as collagen-I, and fibroblast-derived matrix remodeling proteins are essential for mammary developmental and ductal outgrowth [69–71]. As seen in S1c Fig, we performed experiments by implanting cultured mammary gland-derived primary species-matched fibroblasts into cleared MFPs to generate a stromal environment that would be more conducive for ductal formation. First, we confirmed increased collagen deposition and successful engraftment of equine and canine fibroblasts in MFPs that were recovered 2 weeks following implantations by (i) staining with Picro-Sirius (Sirius) red and Masson’s trichrome (MTC) and (ii) performing IHC for vimentin presence using the antibody clone that is non-reactive in mouse [50] (S4 Fig). MFPs that received fibroblast implantations from both species were characterized by dense, collagen-rich populations of vimentin^+^ stromal fibroblasts (Fig 7a), indicating that implanted fibroblasts successfully engrafted and deposited collagen. As expected, these dense regions were absent in MFPs that received the Matrigel vehicle injections (Fig 7a). Second, we xenotransplanted equine and canine mammary gland tissue fragments 2 weeks after the species-matched fibroblast implantations and analyzed the xenografts 9 weeks later (S1c Fig). In contrast to previous reports with immortalized human fibroblast pre-implantations [29, 38], we did not observe ductal growth in equine or canine xenografts in the presence of species-matched fibroblast engraftment prior to mammary xenotransplantations, as assessed by acetocarmine staining of wholemounts. Specifically, equine mammary xenografts within fibroblast pre-implanted MFPs showed a lobule-like morphology confined to the xenotransplantation site, which was similar to wholemounts that received vehicle injections (Fig 7b) or no pre-implantation procedure at all (Fig 2a). Despite a similar overall lobule-like morphology, it was noticed that some equine xenografts showed more blebbing formations after fibroblast pre-implantation (< 20% of xenografts) when compared to vehicle control (0% of xenografts in the vehicle group) (Fig 7b). The morphology of the canine xenografts was also lobule-like in both fibroblast pre-implanted and vehicle groups, with interconnected outgrowth structures observed (Fig 7b). Both vehicle and fibroblast pre-implanted groups displayed similar proportions of blebbing formations (~35–50% for both groups), indicating that this morphology in canine xenografts may be a species-specific characteristic. When quantifying 2D surface areas, there were no statistically significant differences between treatment groups for both species, despite a trending increase in surface area in the fibroblast pre-implanted groups (Fig 7c).

Due to the trending increase in xenograft surface area, an *in vitro* experiment using a BrdU-ELISA assay was performed as a proxy to assess the capacity of paracrine signaling by primary mammary fibroblasts to drive mammary epithelial cell proliferation. Mammosphere-derived epithelial cells (MDEC), which are heterogenous cultures of primary mammary cells derived through the same approach used to generate mammary tissue fragments for xenotransplantation surgeries [43], were treated with species-matched primary fibroblast conditioned media (CM) and subjected to the BrdU-ELISA to determine changes in mammary cell proliferation (Fig 7d). Interestingly, MDEC cultures treated with primary mammary fibroblast CM showed decreased proliferation when compared to MDEC cultures treated with control base media (DMEM), suggesting that paracrine signaling from pre-implanted mammary fibroblasts is most likely not a major driver of mammary xenograft growth *in vivo*.

## Discussion

Mammary gland function (i.e., lactation strategies), and disease incidence (i.e., mammary cancer) vary drastically across mammals [4, 72]. These variations allow for a unique comparative species approach to identify novel regulatory factors and underlying molecular mechanisms of mammary gland (patho)physiology that are relevant to both human and veterinary health. Such research, however, is challenging due to logistical constraints and limited model availability, especially for large and rare mammals, respectively. Thus, a more manageable *in vivo* model is warranted. Here, we describe a reproducible and functional xenotransplantation model that uses cryopreserved mammary tissue fragments from equine or canine donors. Characteristics such as bilayered architecture, vascularization, supportive fibrous stroma, milk production, and hormone responsiveness in mammary xenografts demonstrate the generation of functional mammary structures. Importantly, we used minimally processed cryopreserved tissue fragments containing both stroma and parenchyma, which overcomes the need for immediate access to fresh tissues and allows for long-term storage of mammary gland tissue fragments of virtually any mammal, especially those that are wild and/or rare.

While the overall features were similar for equine and canine donors, several differences were noted. Specifically, when demonstrating xenograft functionality by inducing pregnancy in host mice, we found an upregulation of lactation-associated genes (*CSN2* and *LGB1*) and β-LG presence within xenografts for both donor mammals recovered from pregnant mice, whereas an increased proliferative index was observed in canine, but not equine xenografts. During host pregnancy, there was a mild increase in the presence of inspissated eosinophilic deposits that were evident solely in equine, but not canine, xenograft tissue sections. This discrepancy could be due to innately different responses to circulating hormone concentrations between these two donor species, particularly when considering species-specific differences in circulating pregnancy hormone levels and gestation [66–68, 73], which may not be reflected adequately in the host mice at 18 dpc. Notably, the length of pregnancy varies across all three mammals, with ~20 day [74], ~ 62 day [75], and ~350 day [76] gestation periods for mice, dogs, and horses, respectively, indicating that pregnancy hormone-associated temporal signaling differs across mammals. Furthermore, circulating pregnancy hormone concentrations differ in the two donor species compared to mice. For example, the serum concentration of 17β-estradiol (E2), a potent estrogen that promotes post-natal mammary growth, increases during pregnancy [77, 78] and rises to ~ 35 pg/ml in dogs and ~ 50 pg/ml in horses at 2 weeks and 5 months gestation, respectively [79–81], but rises to a substantially greater concentration of ~ 1000–1800 pg/ml in mice at 7 days gestation [68]. Interestingly, administration of E2 in cows promotes mammary involution and decreases milk synthesis [82], indicating that high E2 levels within host mice may have potentially had an inhibitory effect on the pregnancy-associated phenotypes in the equine xenografts. Furthermore, prolactin, a hormone essential for mammary alveolar development [83], differs structurally in equine relative to multiple other mammals by the number of cysteine residues [84–86], indicating potential challenges with cross-species signaling. To address these hormonal differences, ectopic hormone stimulation, administered either via injection [26] or subcutaneous implantations of slow-release hormone pellets [26, 28, 87], that more accurately reflects hormonal concentrations and timepoints observed during donor mammal pregnancies may be required to better accentuate xenograft growth and development [28]. This would be particularly relevant for future studies intending to investigate variations in mammary lobule development and lactation. Despite these discrepancies across mammals, the findings demonstrate that both equine and canine mammary xenografts are hormonally responsive.

Mammary xenografts from both donor mammals displayed bilayered epithelial architectures that recapitulate the donor gland, however, these structures were confined to the xenotransplantation site and lacked extensive ductal branching. In an attempt to initiate mammary ductal formation, we pre-implanted species-matched primary mammary fibroblasts into the cleared MFPs 2 weeks prior to mammary tissue fragment xenotransplantation. Using this approach, we intended to introduce factors important for ductal morphogenesis, such as extracellular matrix (ECM) remodeling, which promote fibroblast-derived growth factor signaling [29, 38, 69]. Unlike findings that observed immortalized fibroblast-driven mammary duct formation in human xenografts [38], our approach did not drive ductal morphogenesis in either equine or canine xenografts, with xenografts across treatment groups retaining a lobule-like morphology. Interestingly, an infrequent incidence of structural blebbing was observed in equine xenografts following fibroblast pre-implantation, as well as in both vehicle-treated and fibroblast pre-implanted canine xenograft groups. These observations may be a result of species-specific variations in donor fragments, variations in response to host-derived growth factors/stromal components, and/or responses to species-matched fibroblast or Matrigel-derived ECM components/growth factors. Furthermore, it is possible that fibroblast pre-implantation promotes the growth of the mammary xenografts, as indicated by overall increased 2D surface area in the fibroblast pre-implanted groups, however these analyses did not reach statistical significance. Since the pre-implanted primary mammary fibroblasts were observed in dispersed patches throughout the MFPs, these cells may not have been in direct contact with xenotransplanted mammary fragments, thus, we investigated whether paracrine signals from primary mammary fibroblast can drive mammary epithelial cell proliferation in a controlled *in vitro* setting using primary mammosphere-derived epithelial cell (MDEC) cultures. Interestingly, and in contrast to other reports assessing fibroblast CM stimulation in mammary epithelial cell cultures [88, 89], treatment of MDEC cultures with primary mammary fibroblast CM resulted in decreased cell proliferation. However, studies where primary fibroblasts were co-cultured with mammary epithelial cells reported decreased mammary cell proliferation [70, 90], indicating that mammary epithelial cell-fibroblast interactions are not always pro-proliferative, and thus, it is possible that direct signaling and/or ECM remodeling, but not paracrine signaling, by mammary fibroblasts, is the major driver for inducing xenograft ductal branching in this our model. To evaluate whether pre-implanted fibroblasts have some effects on the xenografts despite a lack of ductal morphogenesis, we could in future studies assess the expression of genes associated with ductal morphogenesis, such as transforming growth factor beta 1 (TGFβ-1) [91], or determine if cell polarity at apical regions of the mammary xenografts is altered, as previously reported [70, 92]. Also, further optimization of the fibroblast implantation process, potentially via fibroblast irradiation, fibroblast immortalization, or generation of transgenic fibroblast populations with expression profiles that promote fibroblast MFP colonization and/or ductal morphogenesis [29, 38, 69], may drive ductal formation in equine and/or canine xenografts. Following insights gained from mammary ductal morphogenesis experiments in human mammary organoids, one approach could be to encapsulate mammary fragments within collagen gels that reflect donor stroma collagen content prior to xenotransplantation [93, 94]. Furthermore, the addition of an inhibitor of the Rho-ROCK-myosin II signaling cascade has been shown to drastically increase organoid ductal branching, and thus, incorporating this inhibitor within an encapsulating collagen gel may be a promising approach to improve xenograft branching morphogenesis [93, 94]. Nevertheless, and despite a lack of extensive ductal morphogenesis, the xenotransplantation model at its current stage remains suitable to study lobule-related aspects of mammary health and disease *in vivo*.

Despite the fact that equine and canine mammary xenografts recapitulated stromal and parenchymal compartments, there are some limitations of this model. Firstly, xenograft viability was demonstrated up to only 9 weeks, thus, long-term viability of these structures must be considered and future studies assessing long-term xenograft survival and viability are warranted. One study demonstrated that human mammary xenografts in athymic nude mice were detectable at 25 weeks post-surgery, although they were present at a significantly lower frequency compared to 4 weeks post-surgery [28]. Compared to NSG murine hosts, athymic nude mice are relatively immunologically competent, thus, NSG mice are considered more permissive to tissue engraftment [95] and may be more suited to long-term xenograft analyses compared to athymic nude hosts. Secondly, given that the xenografts lack drainage architecture, some xenografts exhibit a ductal ectasia-like phenotype [96], i.e., the accumulation of proteinaceous fluid within dilated lumina, which results in inflammation and fibrosis under normal immunologic conditions [97]. To this end, effectively inducing xenograft duct formation may be particularly crucial to reduce proteinaceous buildup and alleviate this ectasia-like phenotype. Lastly, given that the NSG hosts are immunodeficient, experiments using this model do not account for the regulatory roles of the immune system in mammary health and disease [98–100]. Despite these limitations, this model still allows for the *in vivo* assessment of stromal/epithelial cell responses to treatments (e.g., carcinogens), indicating value for cell- or compartment-specific analyses.

One of the reasons for selecting equine and canine mammary tissue fragments was based on their natural variation in mammary cancer incidences, despite both domesticated mammals sharing similar habitats with humans [4, 7]. Our group has published work describing molecular mechanisms that might potentially drive this difference in cancer incidence using *in vitro* primary cell cultures from these two, and other, mammals [33, 36]. With the successful generation of equine and canine xenografts, we are now able to confirm and extend this work *in vivo*. For example, and similar to induced carcinogenesis studies in rodent models, host mice containing equine and canine mammary xenografts could be treated with progestins (e.g., medroxyprogesterone acetate), followed by exposure to mammary carcinogens (e.g., 7,12-dimethylbenz(a)anthracene (DMBA) [101–103] or high levels of estrogens (e.g., 17-β-estradiol) [5, 13, 96], and their xenografts could then be assessed for neoplastic features or other indications of early induced malignancy at the molecular level (S6 Fig). Additionally, host mice containing mammary xenografts may also be adequate models for assessing variations in lactation strategy and developmental processes via hormone supplements or relevant pharmaceuticals (S6 Fig). Given that xenografts have on average a surface area of ~1 mm^2^, they are of adequate size for single-cell RNA sequencing and/or spatial transcriptomics analysis [104] (S6 Fig). Furthermore, current bioinformatics classification tools, such as Xenome [105] and XenoCell [106], providing the opportunity to discriminate host from xenograft sequences, thus allowing for accurate assessment of species-specific expression *in vivo*.

## Supporting information

S1 Fig Timelines for surgical procedures. **(a).** Timeline of baseline (no additional interventions) xenotransplantation procedure. **(b).** Timeline of procedure for mice that were mated to assess xenograft functionality within pregnant hosts. **(c).** Timeline of procedure for mice that received pre-implanted primary mammary fibroblasts prior to mammary xenotransplantation surgeries.
(TIF)

S2 Fig Histological characteristics of equine and canine donor mammary glands. **(a).** H&E staining of equine and canine mammary gland images showing typical mammary structure. **(b).** IHC analyses of β-lactoglobulin (β-LG) expression **(i)** and α-smooth muscle actin (α-SMA), cytokeratin-14 (CK14), cytokeratin-18 (CK18) and estrogen receptor-α (ERα) **(ii)** in equine and canine mammary glands. Arrowheads indicate positive IHC labeling (red colorimetric indicator). Scale bars = 100 μm.
(TIF)

S3 Fig Raw gel images. Uncropped images of 2% agarose gels used to assess the presence of equine or canine gDNA within host mouse MFPs to confirm species identity. Cropped images are presented in Fig 2d.
(TIF)

S4 Fig Vimentin antibody clone (VIM 3B4) species-specific reactivity. IHC analysis of vimentin (clone VIM 3B4) on murine and equine skin epithelium and mammary glands. Scale bar = 100 μm.
(TIF)

S5 Fig NOD scid gamma (NSG) host mouse pregnancy. **(a).** ~ 12-week-old female NSG mice containing mammary xenografts, with a nonpregnant (virgin) mouse on the left and a pregnant (18 days post-coitus, dpc) mouse on the right. **(b).** Acetocarmine-stained whole mount images of the murine mammary gland within the 3^rd^ inguinal mammary fat pads of nonpregnant virgin (left) and pregnant at 18 dpc (right) NSG mice.
(TIF)

S6 Fig Proposed uses for the mammary xenotransplantation mouse model. Diagram depicting proposed research uses for xenografts derived from large mammalian donors to assess research questions in vivo and potential readouts to facilitate downstream analysis.
(TIF)

S1 Video Three-dimensional wholemount video of an equine mammary xenograft demonstrating xenograft structure and vascularization (separate file). Equine mammary xenograft cleared with Clear Unobstructed Brain Imaging Cocktails (CUBIC) and imaged using a light sheet microscope to determine morphology, acini presence, and vascularization. Autofluorescence was used to determine structural morphology (imaged using 561 nm laser). Video shows three-dimensional z-stack taken at individual focal planes imaged every 10 μm. Scale bar = 500 μm.
(MOV)

S2 Video Three-dimensional wholemount video of a canine mammary xenograft demonstrating xenograft structure and vascularization (separate file). Canine mammary xenograft cleared with Clear Unobstructed Brain Imaging Cocktails (CUBIC) and imaged using a light sheet microscope to determine morphology, acini presence, and vascularization. Autofluorescence was used to determine structural morphology (imaged using 561 nm laser). Video shows three-dimensional z-stack taken at individual focal planes imaged every 10 μm. Scale bar = 500 μm.
(MOV)

S3 Video Alternative three-dimensional wholemount video of an equine mammary xenograft demonstrating xenograft structure and vascularization (separate file). Video showing an equine xenograft with discontinuous acini and a bright autofluorescent blood vessel that has penetrated throughout the xenograft. Equine mammary xenograft cleared with Clear Unobstructed Brain Imaging Cocktails (CUBIC) and imaged using a light sheet microscope using autofluorescence to determine structural morphology (imaged using 561 nm laser). Video shows three-dimensional z-stack taken at individual focal planes imaged every 10 μm. Scale bar = 500 μm.
(MOV)

S1 Table Antibodies used for immunohistochemistry analyses. (DOCX)

S2 Table Primers used for PCR-based species identification and gene expression analyses. (DOCX)

S3 Table Xenograft engraftment frequency as determined by histological analyses. (DOCX)
