# Supplementary figures and images for: A xenotransplantation mouse model to study physiology of the mammary gland from large mammals
Source: PLoS One. 2024 Feb 28;19(2):e0298390. doi: 10.1371/journal.pone.0298390 (PMC10901318; doi:10.1371/journal.pone.0298390)

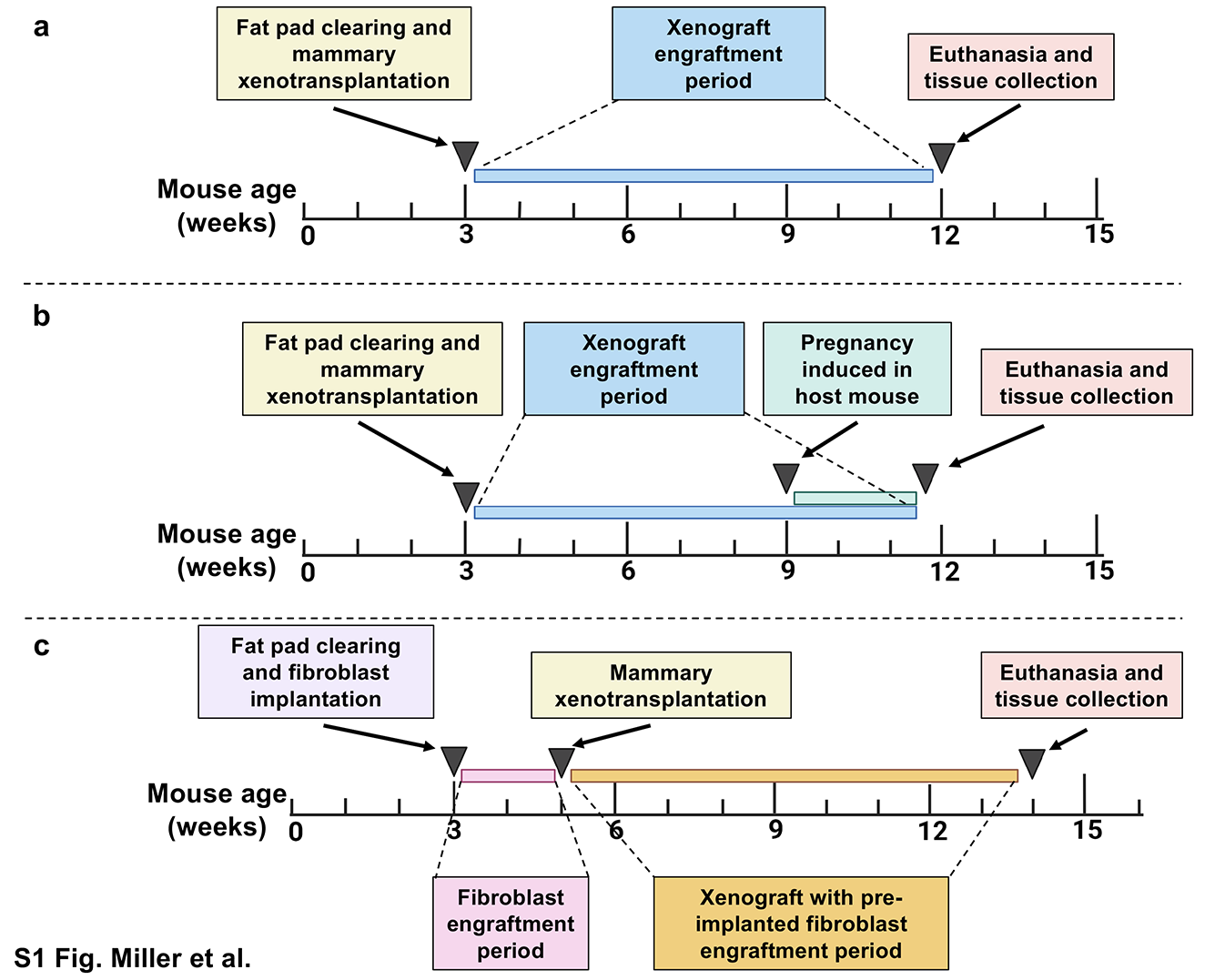

Supplement: S1 Fig — (a). Timeline of baseline (no additional interventions) xenotransplantation procedure. (b). Timeline of procedure for mice that were mated to assess xenograft functionality within pregnant hosts. (c). Timeline of procedure for mice that received pre-implanted primary mammary fibroblasts prior to mammary xenotransplantation surgeries. (TIF) [file pone.0298390.s001.tif]

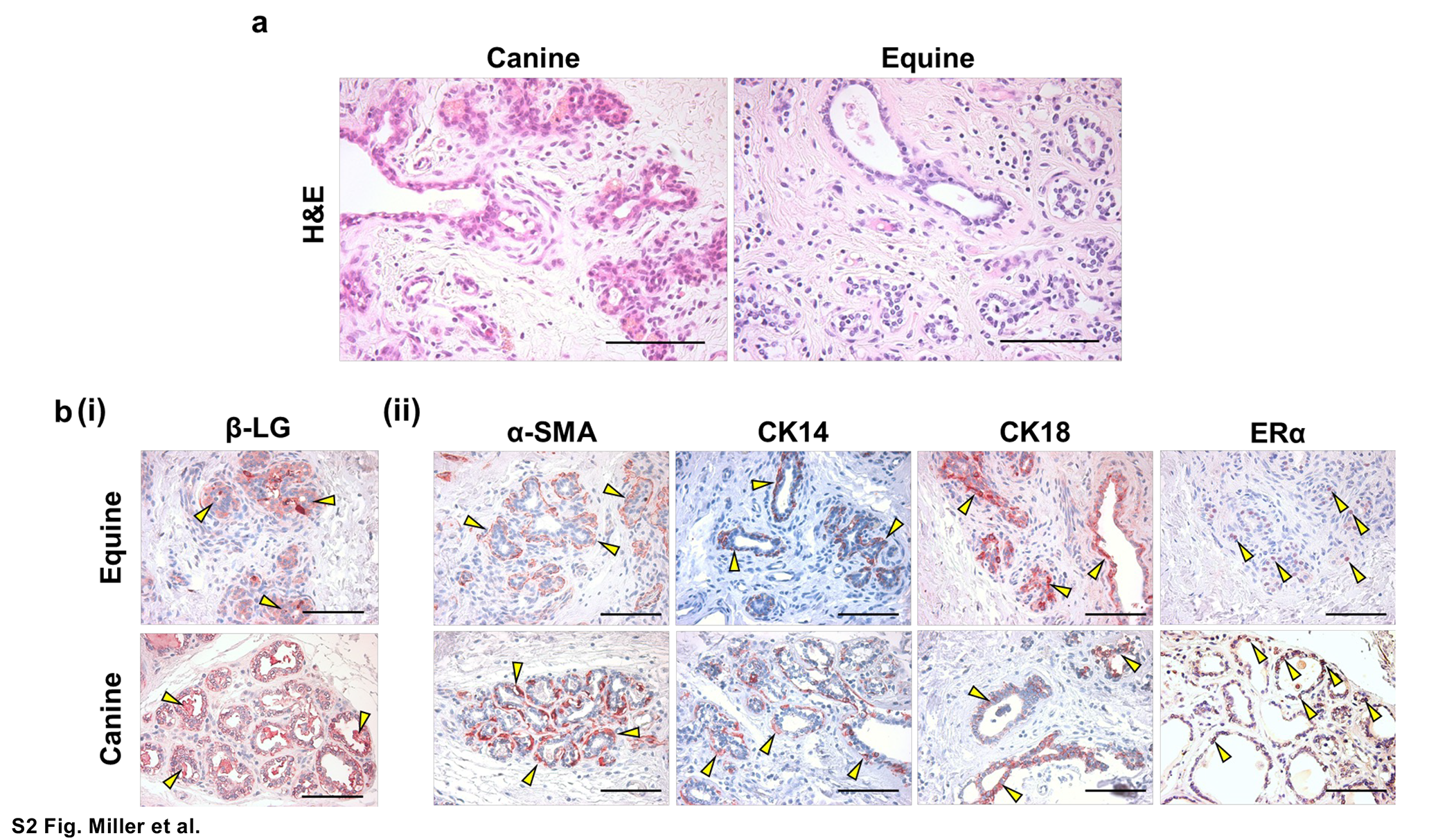

Supplement: S2 Fig — (a). H&E staining of equine and canine mammary gland images showing typical mammary structure. (b). IHC analyses of β-lactoglobulin (β-LG) expression (i) and α-smooth muscle actin (α-SMA), cytokeratin-14 (CK14), cytokeratin-18 (CK18) and estrogen receptor-α (ERα) (ii) in equine and canine mammary glands. Arrowheads indicate positive IHC labeling (red colorimetric indicator). Scale bars = 100 μm. (TIF) [file pone.0298390.s002.tif]

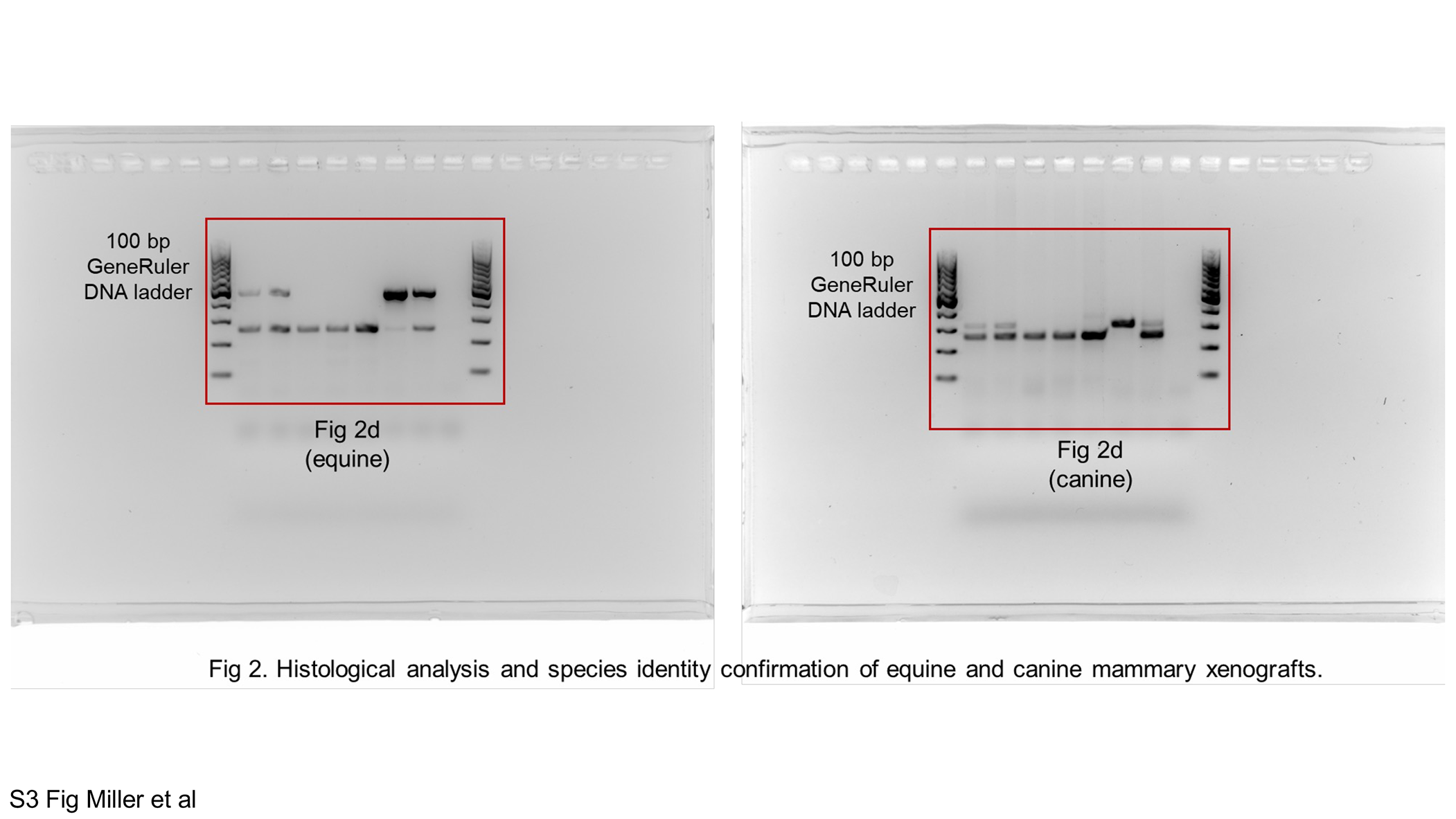

Supplement: S3 Fig — Uncropped images of 2% agarose gels used to assess the presence of equine or canine gDNA within host mouse MFPs to confirm species identity. Cropped images are presented in Fig 2d. (TIF) [file pone.0298390.s003.tif]

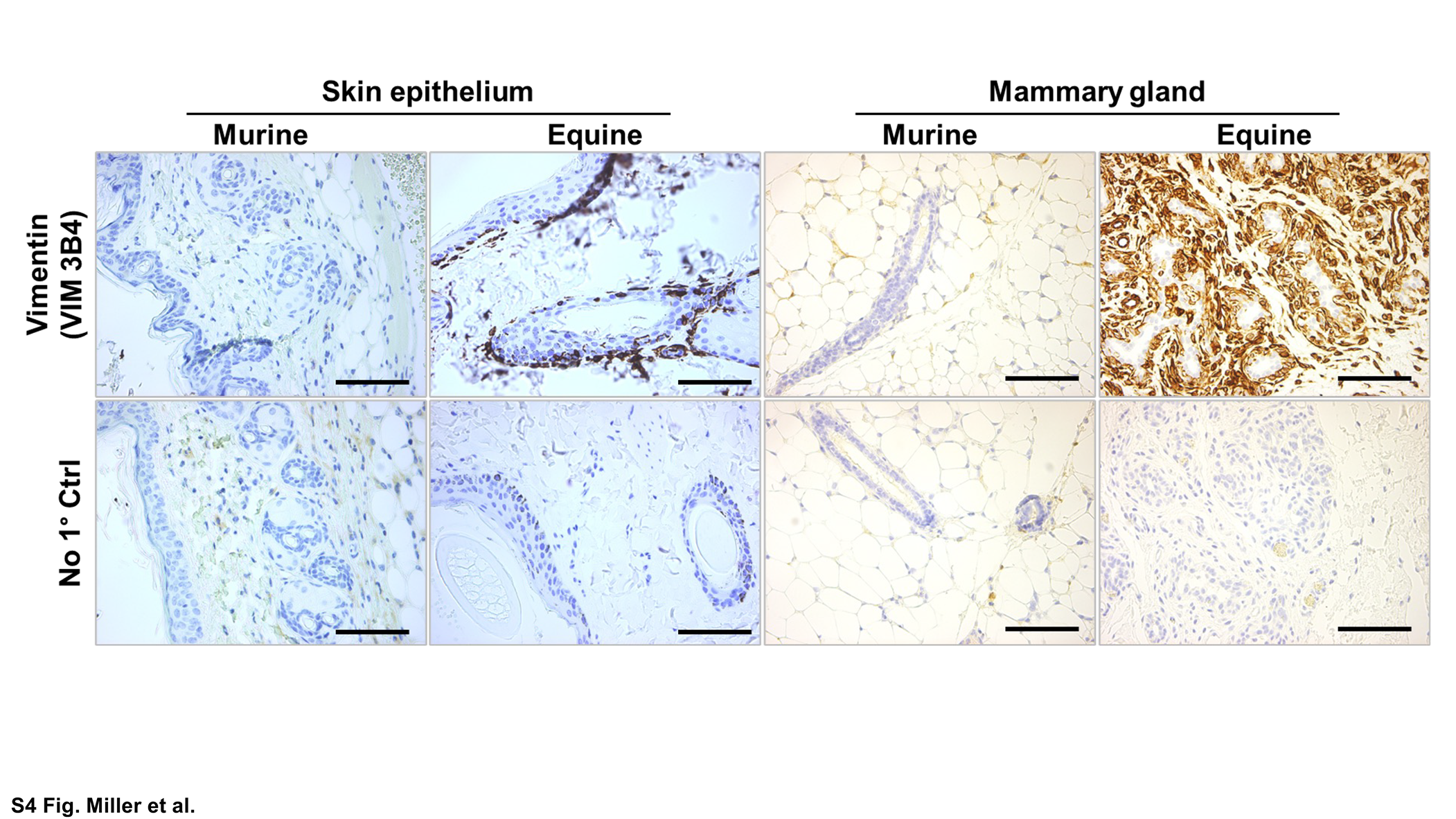

Supplement: S4 Fig — IHC analysis of vimentin (clone VIM 3B4) on murine and equine skin epithelium and mammary glands. Scale bar = 100 μm. (TIF) [file pone.0298390.s004.tif]

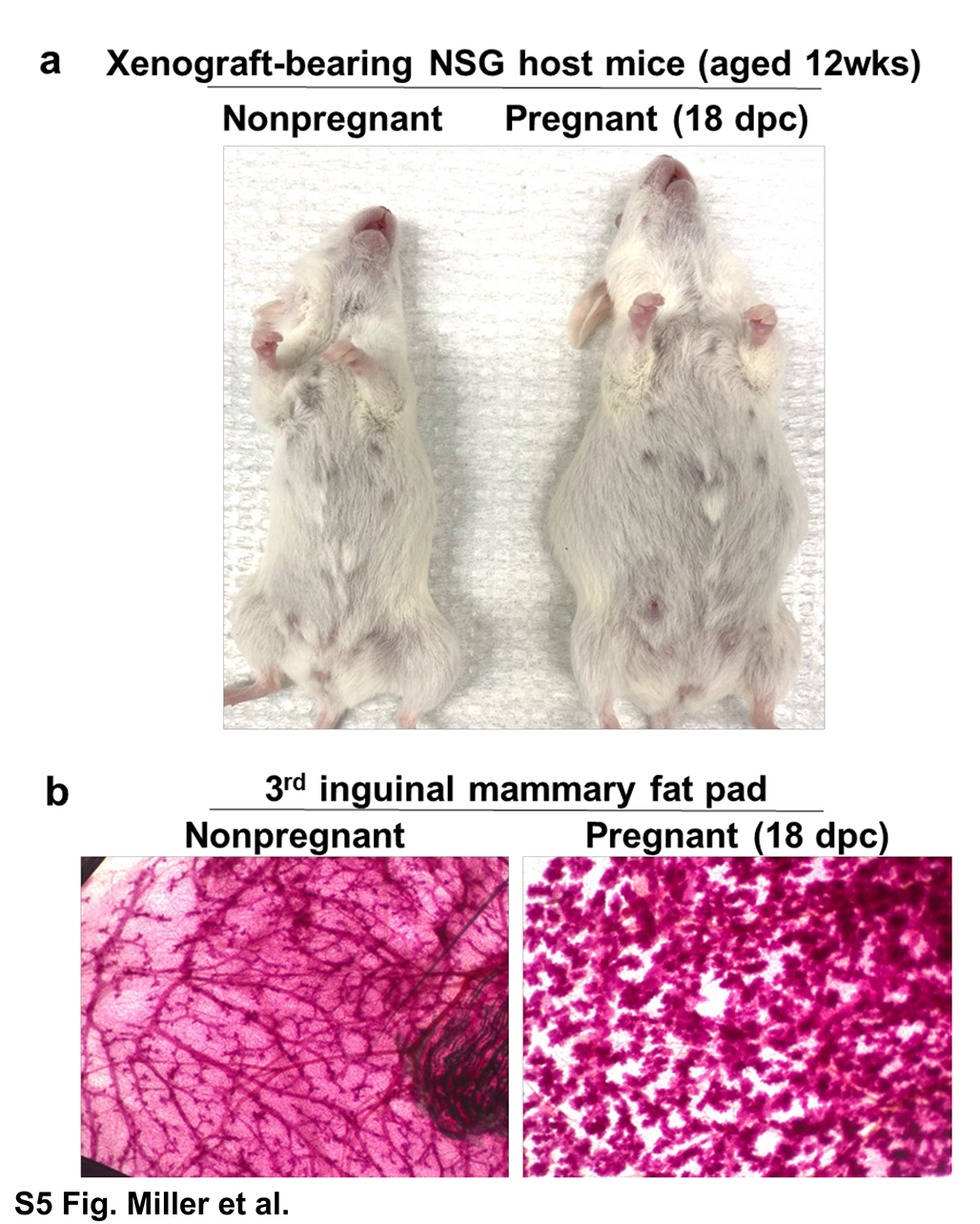

Supplement: S5 Fig — (a). ~ 12-week-old female NSG mice containing mammary xenografts, with a nonpregnant (virgin) mouse on the left and a pregnant (18 days post-coitus, dpc) mouse on the right. (b). Acetocarmine-stained whole mount images of the murine mammary gland within the 3rd inguinal mammary fat pads of nonpregnant virgin (left) and pregnant at 18 dpc (right) NSG mice. (TIF) [file pone.0298390.s005.tif]

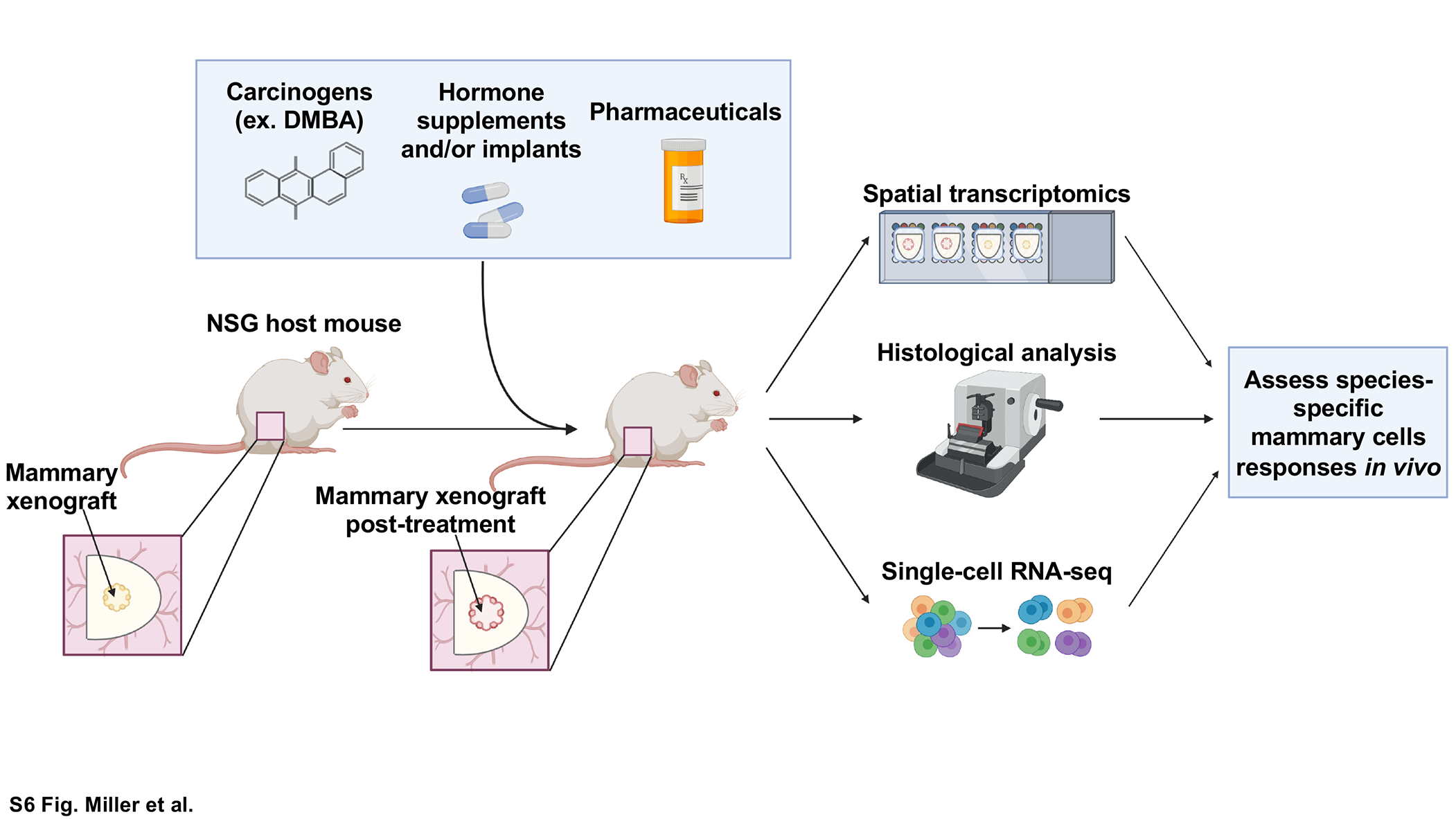

Supplement: S6 Fig — Diagram depicting proposed research uses for xenografts derived from large mammalian donors to assess research questions in vivo and potential readouts to facilitate downstream analysis. (TIF) [file pone.0298390.s006.tif]
