# Supplementary material for: A xenotransplantation mouse model to study physiology of the mammary gland from large mammals
Source: PLoS One. 2024 Feb 28;19(2):e0298390. doi: 10.1371/journal.pone.0298390 (PMC10901318; doi:10.1371/journal.pone.0298390)
Supplement: S1 Table — (DOCX) [file pone.0298390.s010.docx]

**S1 Table. Antibodies used for immunohistochemistry analyses.**

| **Antigen** | **Antibody** | **Dilution** | **Manufacturer** | **Cat #** |
| --- | --- | --- | --- | --- |
| **Primary Antibodies** | |  |  |  |
| Vimentin | Mouse monoclonal, clone VIM 3B4 | 1:80 | Dako | CBL202 |
| β-lactoglobulin (β-LG) | Rabbit polyclonal | 1:200 | Abcam | ab112893 |
| Cytokeratin-14 (CK14) | Mouse monoclonal, clone LL002 | 1:100 | Abcam | ab7800 |
| Cytokeratin-18 (CK18) | Mouse monoclonal, clone C-04 | 1:100 | Abcam | ab668 |
| α-Smooth Muscle Actin (α-SMA) | Mouse monoclonal, clone 1A4 | 1:100 | Dako | M0851 |
| Estrogen receptor-α (ERα) (canine) | Mouse monoclonal, clone TE111.5D11 | 1:25 | ThermoFisher | MA1-12692 |
| Estrogen receptor-α (ERα) (equine) | Mouse monoclonal, clone 6F11 | 1:40 | Abcam | ab93021 |
| Marker of proliferation Ki-67 (Ki67) | Mouse monoclonal, clone MIB-1 | 1:50 | Dako | M7240 |
| Phalloidin-iFluor 488 Reagent | / | 1:500 | Abcam | ab176753 |
| Isotype (IgG) control | Mouse | N/A^a^ | Abcam | ab18443 |
| Isotype (IgG) control | Rabbit | N/A^a^ | Abcam | ab172730 |
| **Secondary Antibodies** | |  |  |  |
| Anti-Mouse IgG (H+L), HRP conjugated | Goat | 1:500 | Jackson | 115-035-062 |
| Anti-Rabbit IgG (H+L), HRP conjugated | Goat | 1:500 | Jackson | 111-035-144 |
| Anti-Mouse IgG (H+L), Biotin-SP | Goat | 1:500 | Jackson | 115-065-166 |
| Anti-Rabbit IgG (H+L), Alexa Fluor 488 | Goat | 1:500 | Jackson | 111-545-144 |
| Streptavidin, HRP conjugated | / | 1:1000^b^/1:250^c^ | Jackson | 016-030-084 |

^a^Isotype control antibody concentration adjusted to match primary antibody concentration on a case-by-case basis. ^b^Streptavidin, HRP-conjugated concentration used in conjunction with Anti-Mouse IgG (H+L), Biotin-SP to boost CK14 and CK18 immunolabelling in equine and canine mammary tissue. ^c^Streptavidin, HRP-conjugated concentration used in conjunction with Anti-Mouse IgG (H+L), Biotin-SP to boost ERα immunolabelling in equine tissue.
