# Supplementary material for: A xenotransplantation mouse model to study physiology of the mammary gland from large mammals
Source: PLoS One. 2024 Feb 28;19(2):e0298390. doi: 10.1371/journal.pone.0298390 (PMC10901318; doi:10.1371/journal.pone.0298390)
Supplement: S2 Table — (DOCX) [file pone.0298390.s011.docx]

**S2 Table. Primers used for PCR-based species identification and gene expression analyses.**

|  |  | **Sequence** | |
| --- | --- | --- | --- |
| **Gene** | **Abbreviation** | **Forward (5’→3’)** | **Reverse (5’→3’)** |
| **gDNA PCR** | |  |  |
| Secretory Carrier Membrane Protein 3 (murine) | *Scamp3* | GAGCTTGACAATCCCTTTCA | TGTTCCCAACATTTGCCTTC |
| Beta-2-Microglobulin (equine) | *B2M* | CGAGACCTCTAACCAGCATC | GCAGAACCATGTCACTCCTC |
| Beta-2-Microglobulin (canine) | *B2M* | CGGAAAGGAGATGAAAGCAG | TTAGTGTTCCCTGCCAATCC |
| **Real-time quantitative (RT-q) PCR** | |  |  |
| Glyceraldehyde-3-phosphate dehydrogenase (equine and canine) | *GAPDH* | ACACCCACTCTTCCACCTTC | TACTCCTTGGAGGCCATGTG |
| β-lactoglobulin (equine) | *LGB1* | CTGCGTGAAGGGGAGAACAA | TAGTCGGTGTCAAGCGCAAA |
| β-lactoglobulin (canine) | *LGB1* | TGCTCGACACGGACTATGAC | GGGCTCTGTTGAATTTCTCC |
| β-Casein (equine) | *CSN2* | CCAGATTCAAGCAGTGAGGAGAA | GGAACGACAGCGTAAGGGAC |
| β-Casein (canine) | *CSN2* | GCAAGAGAGAAGGAAGAACTCAC | TCCTCTCTTTGCTGTTGCTC |
