# Supplementary material for: A xenotransplantation mouse model to study physiology of the mammary gland from large mammals
Source: PLoS One. 2024 Feb 28;19(2):e0298390. doi: 10.1371/journal.pone.0298390 (PMC10901318; doi:10.1371/journal.pone.0298390)
Supplement: S3 Table — (DOCX) [file pone.0298390.s012.docx]

| **S3 Table. Xenograft engraftment frequency as determined by histological analyses.** | | | | |
| --- | --- | --- | --- | --- |
|  | **Equine xenografts** | | **Canine xenografts** | |
| **Experiment** | **Transplanted (#)^b^** | **Observed (#)^a^** | **Transplanted (#)^b^** | **Observed (#)^a^** |
| Baseline^*^ | 16 | 8 | 16 | 9 |
| Pregnant (18 dpc) & non-pregnant comparative analyses | 54 | 28 | 74 | 36 |
| Fibroblast pre-implantation & vehicle treatment comparative analyses | 24 | 12 | 24 | 11 |
| Total | 94 | 48 | 114 | 56 |
| **Overall engraftment success rate (%)** |  | **51.06** |  | **49.12** |

*Xenotransplanted tissues that did not receive additional intervention prior to extraction (baseline). ^a^Number (#) of xenografts observed following histological evaluation of fixed tissues. Evaluation included formalin fixed paraffin embedding (FFPE), sectioning, and hematoxylin & eosin (H&E) staining of serial sections, or whole mount analysis via acetocarmine staining. ^b^Number (#) of mammary tissue fragments associated with each treatment group that were xenotransplanted into host mice. Overall engraftment success rate (%) determined as the number of observed xenografts divided by the total number of mammary tissue fragments used for xenotransplantation.
